# Comparative Study of the Removal Efficiency of Nalidixic Acid by Poly[(4-vinylbenzyl)trimethylammonium Chloride] and N-Alkylated Chitosan through the Ultrafiltration Technique and Its Approximation through Theoretical Calculations

**DOI:** 10.3390/polym15153185

**Published:** 2023-07-27

**Authors:** Daniel A. Palacio, Carla Muñoz, Manuel Meléndrez, Walter A. Rabanal-León, Juliana A. Murillo-López, Manuel Palencia, Bernabé L. Rivas

**Affiliations:** 1 Departamento de Polímeros, Facultad de Ciencias Químicas, Universidad de Concepción, Edmundo Larenas 129, Casilla 160-C, Concepción 4070409, Chile; dapalacio@udec.cl (D.A.P.);; 2 Departamento de Ingeniería de Materiales (DIMAT), Facultad de Ingeniería, Universidad de Concepción, Edmundo Larenas 270, Casilla 160-C, Concepción 4070409, Chile; mmelendrez@udec.cl; 3 Laboratorio de Modelamiento Computacional en Sistemas Inorgánicos y Organometálicos (Lab-MCSIO), Departamento de Química Analítica e Inorgánica, Facultad de Ciencias Químicas, Universidad de Concepción, Edmundo Larenas 129, Casilla 160-C, Concepción 4070409, Chile; 4 Departamento de Ciencias Químicas, Facultad de Ciencias Exactas, Universidad Andrés Bello, Autopista Concepción–Talcahuano 7100, Talcahuano 4260000, Chile; 5 Departamento de Química, Facultad de Ciencias Naturales y Exactas, Grupo de Investigación en Ciencias con Aplicaciones Tecnológicas (GI-CAT), Universidad del Valle, Calle 13#100-00, Cali 25360, Colombia

**Keywords:** nalidixic acid, removal, polymers, ultrafiltration membranes

## Abstract

Emerging antibiotic contaminants in water is a global problem because bacterial strains resistant to these antibiotics arise, risking human health. This study describes the use of poly[(4-vinylbenzyl) trimethylammonium chloride] and N-alkylated chitosan, two cationic polymers with different natures and structures to remove nalidixic acid. Both contain ammonium salt as a functional group. One of them is a synthetic polymer, and the other is a modified artificial polymer. The removal of the antibiotic was investigated under various experimental conditions (pH, ionic strength, and antibiotic concentration) using the technique of liquid-phase polymer-based retention (LPR). In addition, a stochastic algorithm provided by Fukui’s functions is used. It was shown that alkylated *N*-chitosan presents 65.0% removal at pH 7, while poly[(4-vinylbenzyl)trimethylammonium chloride] removes 75.0% at pH 9. The interaction mechanisms that predominate the removal processes are electrostatic interactions, π–π interactions, and hydrogen bonding. The polymers reached maximum retention capacities of 1605 mg g^−1^ for poly[(4-vinylbenzyl) trimethylammonium chloride] and 561 mg g^−1^ of antibiotic per gram for alkylated poly(N-chitosan). In conclusion, the presence of aromatic groups improves the capacity and polymer–antibiotic interactions.

## 1. Introduction

The high contamination of water is one of the main problems faced by humanity. Water is essential for life, food security, health, and well-being. The causes of water contamination and the loss of its safety have their origin in natural and anthropogenic factors, such as pharmaceutical ingredients, endocrine disruptors, and heavy metals, among others [1,2]. In particular, antibiotics are a special class of emerging contaminants capable of inducing adverse effects on ecosystems and health by triggering the emergence of antibiotic-resistant bacteria. As a result, treatments for the control of diseases produced by bacteria lose effectiveness, which puts the patient’s life at risk and increases the pressure and risks of sepsis on the medical and hospital infrastructure [3,4].

Currently, various methods have been described to treat antibiotic-contaminated water, such as adsorption, photocatalysis, chemical oxidation, membrane separation, and biological methods, among others [5,6]. The adsorption technique is characterized by low costs, high removal ability, and easy scaling; however, usually the generation of highly contaminated sludge is a characteristic described as a disadvantage of the technique. Consequently, in many cases, adsorption can be visualized as a previous stage focused on the concentration of pollutants to subsequently carry out a degradation stage, such as photocatalysis, chemical oxidation, or microbiological biodegradation. In contrast, membrane-separation methods can also be described as methods of low cost, high removal ability, and easy scaling, whose main disadvantages are fouling and the processes that are derived from this, as well as the relatively high energy cost depending on the membrane technique used [7,8]. On the other hand, hybrid techniques combine two or more strategies with different removal or separation mechanisms to achieve, through a synergistic approach, greater efficiency in the overall process [9]. In this way, homogeneous phase adsorption is mediated by water-soluble polymers coupled with separation via the use of an ultrafiltration membrane; thus, by this strategy, polymer–pollutant interactions can be carried out in a homogeneous phase, and the selectivity of separation can be improved in a single stage. Some works related to the removal of organic contaminants are as follows: Fradj et al. (2014) used poly(acrylic acid) and poly(ammonium acrylate) for the removal of methylene blue, obtaining high retention values (~98%) under different operation conditions (i.e., ionic strength and pH) [10]; Dasgupta et al. (2015) used polyethyleneimine and chitosan to study the retention effects of an anionic dye (red120), and they reported a retention greater than 99.0% via electrostatic interactions [11]; Palencia et al. (2017) studied the removal of Acid Blue 129 using poly(allyltriethylammonium chloride) and obtained removal values of 95–97% for pH conditions ranging between 4.0 and 12.0 [12]; Arce et al. (2022) described the use of dispersed collagen-based particles for the removal of rhodamine B, achieving a removal of approximately 97% [13]; Oyarce et al. (2021) found values of removal of ~98.0 and ~90.0% for methylene blue and methyl orange using poly(diallyldimethylammonium) and poly(2-acrylamide-2-methyl-1-propanesulfonic acid), respectively [14]. On the other hand, studies have been reported based on the removal of antibiotics. For example, Palacio et al. (2021) obtained three polyelectrolyte copolymers with different charge ratios applied in the removal of ciprofloxacin, obtaining a removal capacity between ~80 and ~90.0% [15]. Palacio et al. (2020) applied alkylated chitosan for the removal of the antibiotics amoxicillin, tetracycline, and ciprofloxacin, obtaining removal capacities of ~70.0% for the three antibiotics [9].

On the other hand, nalidixic acid (or 1-ethyl-1,4-dihydro-7-methyl-4-oxo-1,8-naphthyridine-3-carboxylic acid) is an antibiotic from the group of quinolones that is widely used in both human and veterinary medicine, and at low concentrations, it inhibits bacterial growth and reproduction [16,17,18]. In addition, it has been used to control infections in chicken and aquaculture production systems [19]. Nalidixic acid has been found in water, soil, and especially hospital effluent treatment plants [20]. In this context, the share effect of nalidixic acid removal is evaluated. The LPR technique was used with two cationic polymers with permanent charges and with the presence of aromatic groups (poly[(4-vinylbenzyl) trimethylammonium chloride]) and without aromatic groups (*N*-alkylated chitosan). Furthermore, a computational approach was evaluated in which preferred interaction sites between polymers and nalidixic acid were evaluated using a stochastic search algorithm guided by chemical information provided by the Fukui functions of the interacting fragments.

## 2. Materials and Methods

### 2.1. Reagents and Materials

**Figure 1:**
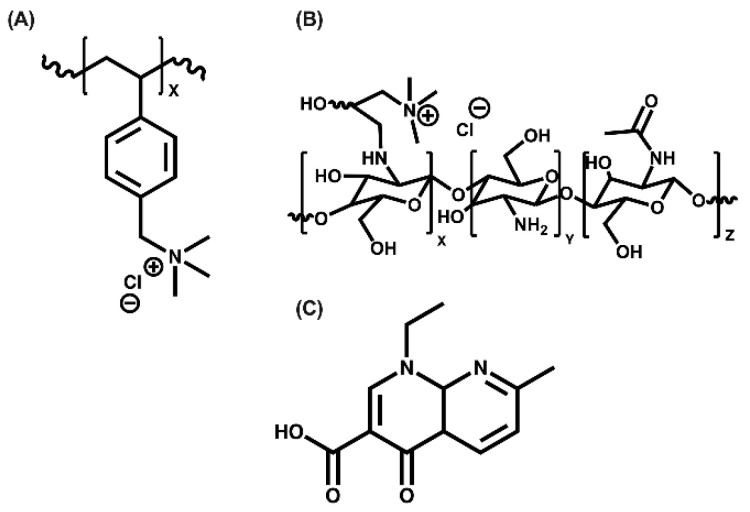
Molecular structure. (**A**) poly[(4-vinylbenzyl) trimethylammonium chloride], (**B**) *N*-alkylation of chitosan, and (**C**) nalidixic acid.

To obtain poly[(4-vinylbenzyl)trimethylammonium chloride] (see Figure 1A), 4-(vinylbenzyl)trimethylammonium chloride was used as a monomer, and ammonium persulfate was used as a radical polymerization initiator. For the *N*-alkylation of chitosan (see Figure 1B), chitosan with a degree of deacetylation of 96.3% and a molecular weight of 646.5 kDa, iodine, *N*-(3-chloro-2-hydroxypropyl) trimethylammonium chloride, sodium hydroxide, acetic acid, and potassium chloride were used. To carry out all the reactions, type I deionized water was used, and for the purifications, dialysis membranes with a molecular weight cut-off size between 12.0 and 14.0 kDa were used (purchased from Sigma Aldrich-Chile). A 100 kDa fraction of poly[(4-vinylbenzyl) trimethylammonium chloride] was used. Nalidixic acid (see Figure 1C) with 98.5% purity was employed for antibiotic-removal assays. All reagents were purchased from Sigma Aldrich-Chile.

### 2.2. Obtaining Polymers and Characterization

#### 2.2.1. N-Alkylated Chitosan

The alkylation process was carried out following the methodology. For this, 5.0 g of deacetylated chitosan was dissolved in a 3% (*w*/*v*) acid solution (CH_3_COOH), and later, a 10% (*w*/*v*) potassium chloride solution was added dropwise with constant stirring. Upon completion, the pH of the solution was adjusted to 9.0 with the help of 15% (*w*/*v*) sodium hydroxide until a gummy solution was obtained, and the supernatant was separated by vacuum filtration. The obtained wet mass was solubilized in 25.0 mL of 60% (*w*/*v*) *N*-(3-chloro-2-hydroxypropyl) trimethylammonium chloride and stirred for a period of 6 h. Subsequently, it was subjected to heating at 90 °C for 24 h with constant stirring. Subsequently, 2.5 g of iodine and 100 mL of 10% sodium hydroxide were added and stirred for another 18 h at the same temperature. For purification, regenerated cellulose bags were used for the dialysis process with water exchange every 6 h. When finished, the bags were frozen and lyophilized for subsequent application [9].

#### 2.2.2. Poly[(4-vinylbenzyl) Trimethylammonium Chloride]

Polymerization was carried out by free-radical polymerization. For this, 11.8 mmol of 4-(vinylbenzyl) trimethylammonium chloride and 1 mol% of initiator, with respect to the mole of monomers used, were dissolved in 20 mL of water. Polymerization was carried out at 80 ± 2 °C for 24 h under a nitrogen atmosphere. For purification of the homopolymer, the product obtained was dissolved in 1 L of distilled water and placed in an ultrafiltration cell with a regenerated cellulose membrane with a molecular weight cut-off size of 100 kDa. All proteins above 100 kDa were selected for further studies, frozen, lyophilized, and stored in airtight containers due to the high hygroscopicity of the material.

The samples obtained in the processes described in Section 2.2.1 and Section 2.2.2 were characterized using an FTIR spectrometer (Perkin Elmer, Waltham, MA, USA) with the total attenuated reflection function (ATR) were used. Each spectrum was obtained by consecutive scans in the range of 4000 to 500 cm^−1^ with a resolution of 1 cm^−1^. Nuclear magnetic resonance (NMR) measurements were obtained using a Bruker Ascend™ 400 MHz spectrometer. The analyses were conducted using D_2_O/CF_3_CO_2_D (1%, *v*/*v*) to dissolve 10 mg of deacetylated chitosan and D_2_O to dissolve 10 mg of *N*-alkylated chitosan and poly[(4-vinylbenzyl) trimethylammonium chloride] and its respective monomer.

### 2.3. Antibiotic-Removal Studies

**Figure 2:**
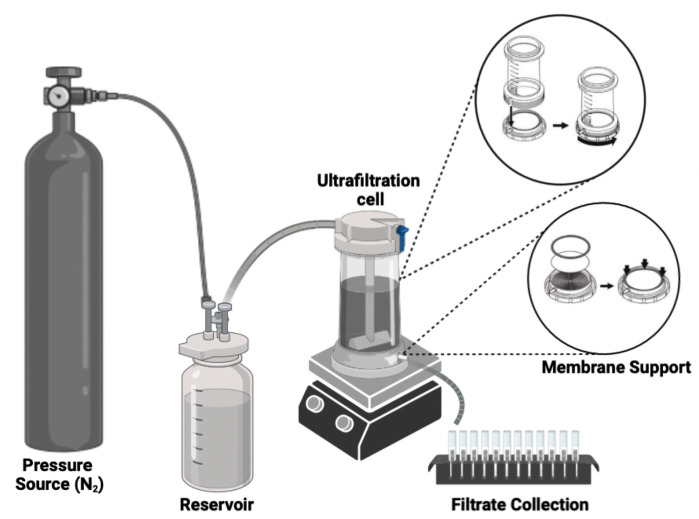
Schematic representation antibiotic-removal studies.

The removal of the antibiotic (R_pol_)was carried out using polymer-enhanced ultrafiltration by the washing method [9] (see Figure 2) using separate aqueous dissolutions of poly[(4-vinylbenzyl) trimethylammonium chloride] and *N*-alkylated chitosan as retaining phases. The effects of variations in pH (i.e., 3.0, 5.0, 7.0, 9.0, and 11.0), ionic strength (i.e., 0.0, 0.01, 0.1, 0.2, 0.5, and 1.0 mol L^−1^ NaCl), and antibiotic concentration (i.e., 5, 10, 20, 100, and 200 mg L^−1^ nalidixic acid) on retention were studied. In addition, the saturation capacity of the polymers and their discharge capacity were evaluated using water at different pH values where the highest retention values were presented [21]. In all cases, 20 mL of permeate was sequentially collected in collecting tubes for a total permeate volume of 200 mL per test. For the saturation studies, concentrations of 43.0 mg L^−1^ and 0.01482 g for poly[(4-vinylbenzyl) trimethylammonium chloride] and 0.0332 g for *N*-alkylated chitosan were used. Discharge tests and polymer recovery were performed at pH 3.0.

For the saturation studies, concentrations of 43.0 mg L^−1^ and 0.01482 g for poly(4-vinylbenzyl) trimethylammonium chloride and 0.0332 for *N*-alkylated chitosan were used. For the discharge, an acidic pH was used to evaluate the elution efficiency of the antibiotic. The quantification of the antibiotic was carried out using a UV–Vis spectrometer (Evolution One Plus, Thermo Fischer SCIENTIFIC, Waltham, MA, USA) at a wavelength of 258 nm scanning between 200 nm and 500 nm.

To compare the experimental data and predict the behavior between the interactions of the polymer and the antibiotic, we apply a mathematical model of ion distribution, knowing that the interactions between the polymers and the antibiotic molecules in aqueous solutions depend mainly on the nature of the interactions [22]. Although there are different variables to consider, we rely purely on the interactions between the polymer and antibiotic, assuming that there is no interference between the membrane and the antibiotic. Knowing this, one can distribute the effects in (1) the volume of the solution (the fraction of the solution in which the antibiotics are not affected by the interaction with some cell-retaining component); (2) the permeate (the fraction of solution that can pass through the membrane and corresponds only to the solvent and the solutes distributed in the volume of the solution); and (3) the domain of the polymer (the region where the counterions strongly interact with the polymer) and the polymer constant ($k_{pol}$), which is given by:

$$C_{p}=\frac{\alpha (1+\theta_{pol})C_{0}}{\alpha (1+\theta_{pol})+F}$$

with:

$$\theta =1+k_{pol}\frac{V_{pol}}{V_{c}}=1+\theta_{pol}$$

$$\alpha =\frac{{dC}_{p}}{{dC}_{c}}\approx constant$$

where *F* is the filtration factor given by the volume of the permeate ($V_{P}$) between the volume of the cell ($V_{C})$; $C_{c}$ is the cell concentration; $C_{p}$ is the concentration of the permeate; $V_{pol}$ is the domain of the polymer; $C_{0}$ is the initial concentration of antibiotic added to the ultrafiltration cell; and $\theta_{pol}$ is defined as the interaction parameter of the system given by the following:

$$\theta_{pol}=1+k_{pol}V_{pol\;}{V_{C}}^{-1}$$

### 2.4. Computational Details

To evaluate the complicated polymer conformation, a reduced model was used to describe polymer chains for both studied systems, *N*-alkylated chitosan (polymer 1) and poly[(4-vinylbenzyl) trimethylammonium chloride] (polymer 2). To achieve this, a polymer length three times the length of the antibiotic (nalidixic acid) was considered to have sufficient extension to evaluate most interaction positions. This construction rule provides a structural dimer for the *N*-alkylated chitosan polymer and a structural hexamer for the poly[(4-vinylbenzyl) trimethylammonium chloride] polymer.

These polymer structures, as well as nalidixic acid, were fully optimized at an initial stage through the use of the PM6 semiempirical method [23], and then these structures were refined at the B3LYP/6-31g(d,p) level of theory [24,25]. The presence of local minima structures was confirmed by means of frequency analysis. All these calculations were carried out using the Gaussian16 program [26].

Once the structural models for both polymers and the antibiotic were obtained, the following step was to evaluate the potential candidate structures to describe the interaction between both polymers and nalidixic acid. To achieve this, the “Kick−Fukui” hybrid method proposed by O. Yañez et al. was employed [27]. This methodology considers a stochastic search of global minima structures via the use of the SnippedKick [28] algorithm but considers the interaction energy term provided by the chemical information included in Fukui’s functions of the interacting fragments [29,30]. To obtain the final candidate structures of each type of polymer–antibiotic interaction, 22,000 structures were evaluated (1000 × N, where N is the total number of atoms in the system), and the number of these structural candidates was reduced by two factors: (i) ranking in terms of the Coulomb integral value of the Fukui function (the highest value suggests the strongest interaction) and (ii) using the Grygorian–Springborg algorithm [31,32,33] to discriminate duplicate structures. These considerations allow us to considerably reduce the number of structures to analyze. For the interactions of nalidixic acid with polymer 1, there were four final structures, and there were three for polymer 2. Each fragment was optimized at the same level of theory as their precursors, and the interaction energies were evaluated. To define the kind of chemical interactions among fragments (polymer–antibiotic), NCI (noncovalent interactions) analysis [34,35,36] was carried out. All the necessary topological information for the Kick–Fukui algorithm was obtained using the TAFF pipeline [36] and the Multiwfn 3.6 code [37].

## 3. Results and Discussion

### 3.1. Characterization of the Polymers

**Figure 3:**
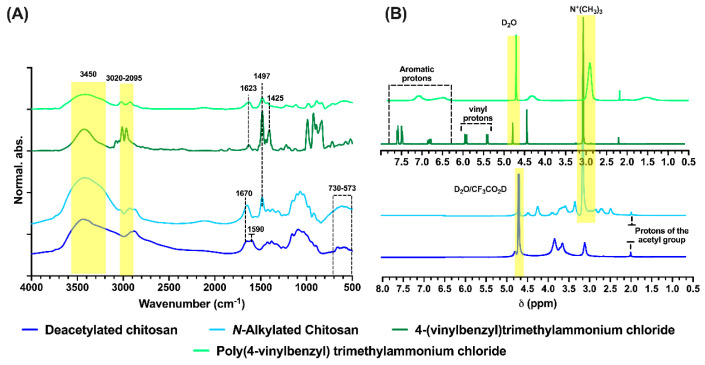
Spectroscopic characterization using (**A**) Fourier-transform infrared spectroscopy with attenuated total reflectance (FTIR-ATR) and (**B**) proton nuclear magnetic resonance (^1^H-NMR).

In Figure 3A, the FTIR spectra of starting polymers and monomers can be seen, such as the polymers obtained for their application. In the case of deacetylated chitosan and N-alkylated chitosan, characteristic absorption bands are observed between 730 cm^−1^ and 573 cm^−1^, characteristic of the acetyl glucosamine groups of the polymer chain, and at 1497 cm^−1^, characteristic of the quaternary ammonium groups. This also confirms the absence of the primary amine band at approximately 1590 cm^−1^, present in deacetylated chitosan, and an adsorption band at 1670 cm^−1^ (characteristic of the primary amide) 3020 cm^−1^, and 2095 cm^−1^, attributed to -CH and -C-C stretching and at 3450 cm^−1^ vibration stress of -NH and -OH. For the monomer and poly[(4-vinylbenzyl) trimethylammonium chloride], there are characteristic absorption bands at 1425 and 1623 cm^−1^ attributed to the C=C bonds of the aromatic ring; at 1497 cm^−1^, characteristic of the presence of quaternary ammonium groups; between 3020 cm^−1^ and 2095 cm^−1^, attributed to -CH and -C-C stretching; and at 3450 cm^−1^ due to the stretching of the -OH groups, which is attributed to the fact that both the monomer and the polymer are highly hygroscopic [38,39].

The NMR results (see Figure 3B) show the characteristic signals of the corresponding spectra for each polymer. For the N-alkylated chitosan, a signal can be observed at approximately 3.1 ppm, attributed to the protons of the amino groups after the alkylation process [40], which is not observed in commercial chitosan with a degree of deacetylation of 96.0%. For poly[(4-vinylbenzyl) trimethylammonium chloride], the characteristic signal of the protons of the quaternary ammonium groups can be observed at approximately 3.1 ppm, with the presence of aromatic protons at approximately 6.5–7.7 ppm and the absence of vinyl protons in chemical displacements between 5.3 to 6.0 ppm, which do appear in the NMR spectrum of the monomer, confirming that the polymerization process was carried out and that it does not present residual monomers.

### 3.2. Antibiotic-Removal Studies

**Figure 4:**
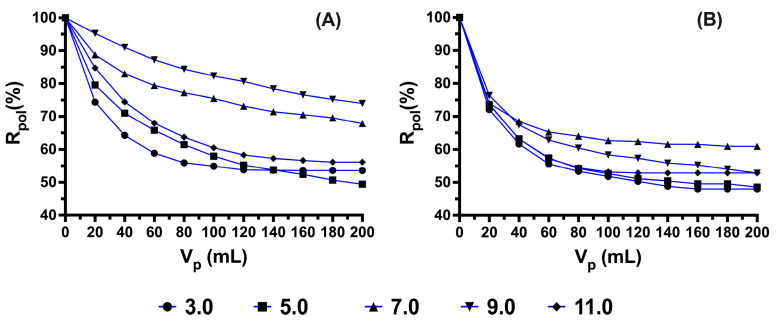
Retention profiles at different pH values: (**A**) poly[(4-vinylbenzyl) trimethylammonium chloride] and (**B**) *N*-alkylated chitosan.

Studies based on pH are essential in the processes and mechanisms of removal between the polymer and the antibiotic because pH is a parameter that allows us to determine the removal efficiency mainly due to acid-base speciation processes of the antibiotic [41], depending on the functional groups present in its structure. The retention results as a function of the variation in the volume of the permeate are shown (see Figure 4). Figure 4A,B shows low percentages of removal at pH 3.0 and an increase in the removal processes as pH values are increased, in addition to a slight decrease in the maximum pH of this study. The effect of pH on removal can be explained by the dissociation of carboxylate groups given that nalidixic acid has a single pKa value of 5.95; consequently, for pH > 5.95, the electrostatic interactions between the polymer and antibiotic are favored [42]. It is worth noting that all the retention effects presented are due to polymer–antibiotic interactions since the membrane does not interfere in the retention processes (see Figure S1).

From the information provided above, the greatest interactions are favored at pH values greater than 5.95, which agrees with the results obtained experimentally and is governed mainly by electrostatic interactions between the quaternary ammonium groups of the polymers and the dissociated carboxylic groups of the antibiotic. It should be noted that polymers at a structural level behave totally differently, which is a factor that plays a role in the increase in retention processes, and retention at a pH less than 5.95 is influenced by low electrostatic interactions due to slightly ionized molecules that can interact with polymers. Additionally, other types of interactions could influence the behavior of the elution curve, such as hydrogen bonds or ***π***–*π* interactions of the aromatic rings of poly[(4-vinylbenzyl) trimethylammonium chloride] and the quinolone-type structure of antibiotics [43,44].

Although these may be slightly affected as the pH values increase, for the pH value where the greatest retention capacity is observed, this may be influenced in the case of *N*-alkylated chitosan due to its ability to curl up at basic pH, which may be influenced to maintain their retention processes. However, in general, the two polymers under study can also be affected by an increase in ions that make the polymer chains less conformationally available so that the functional groups can interact with the antibiotic molecules and slightly decrease the removal percentages.

**Figure 5:**
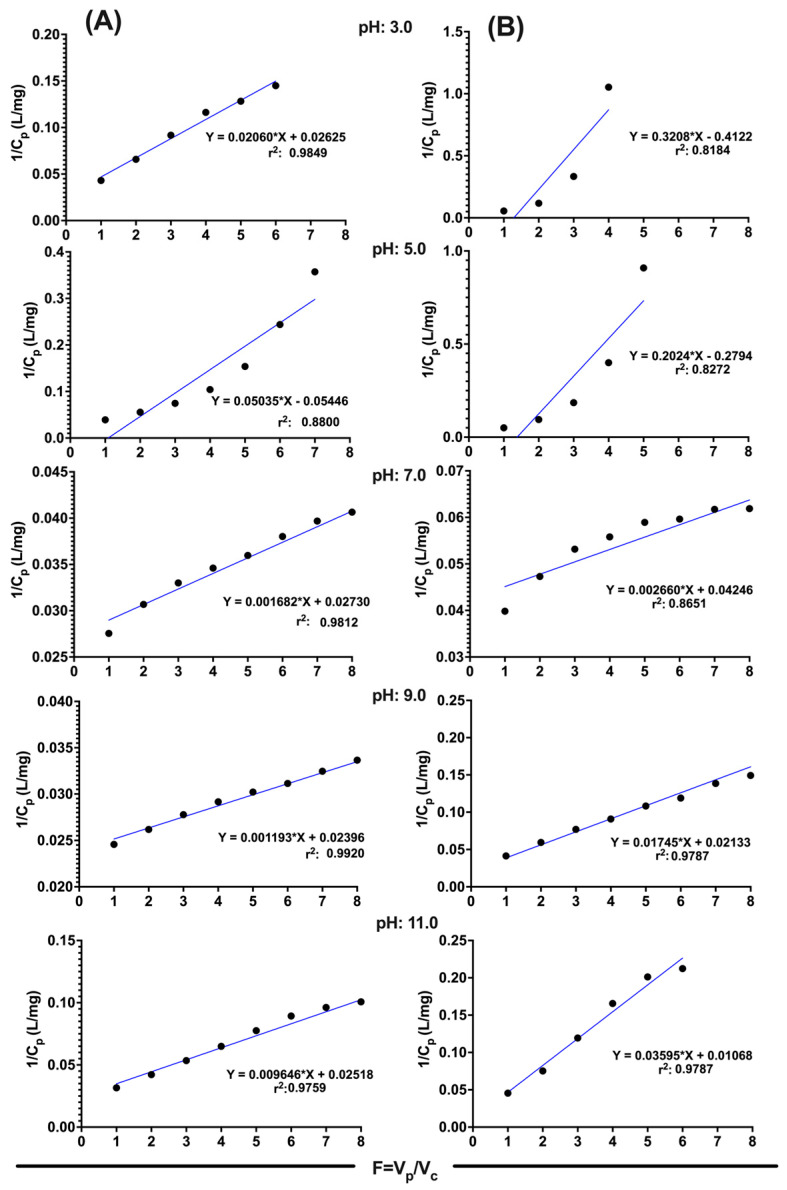
Experimental data models of retention profiles at different pH values: (**A**) poly[(4-vinylbenzyl) trimethylammonium chloride] and (**B**) *N*-alkylated chitosan.

Based on the mathematical formula of the previously proposed model, the experimental data are linearized by plotting 1/Cp vs. F for the two polymer systems and the pH studied (see Figure 5). From the results obtained, we can predict through its correlation coefficient the adjustments of the experimental data correlated with adequate values of Co [12,22]. Based on these results, we can confirm that the best interactions are carried out at pH 9.0 for both polymers, which correlates with the results of retention percentages of poly[(4-vinylbenzyl) trimethylammonium chloride]. However, for N-alkylated chitosan, the highest retention percentages were observed at pH 7.0, and this slight disparity may be influenced by the structural characteristics and conformational behavior of the polysaccharide chains.

**Figure 6:**
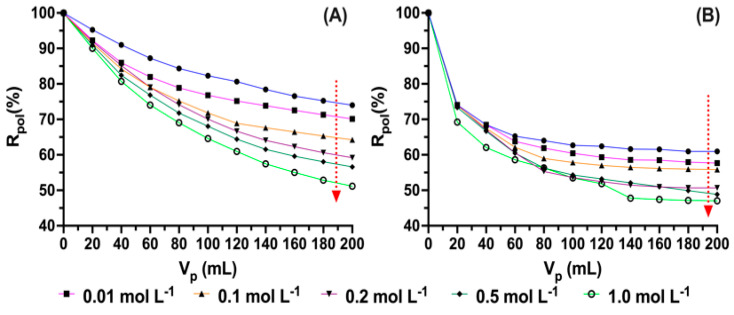
Retention profiles at different ionic strengths: (**A**) poly(4-vinylbenzyl) trimethylammonium chloride (pH: 9.0) and (**B**) *N*-alkylated chitosan (pH: 7.0).

Figure 6 shows the results obtained as the concentration of ionic strength increases. As the concentration of sodium chloride increases, the percentage of antibiotic removal decreases for the two systems under study. These processes of low retention as the concentrations of ions in solutions increase may be mainly due to the competition of ions for the active sites of the chains and a balance between the availability of ions in solutions and the speciation of the molecules of antibiotics, as by the active sites of the polymer. In the same way, some authors report that high counterion contents produce the generation of ionic bridges between the available groups, which causes the chains to be more extended or coiled. This is associated with the fact that the conformation of the polymer can be slightly affected by the presence of ions in the solution due to high interactions or the presence of repulsive forces that cause the polymer structure to be slightly affected and decrease interactions with the analyte [45,46].

**Figure 7:**
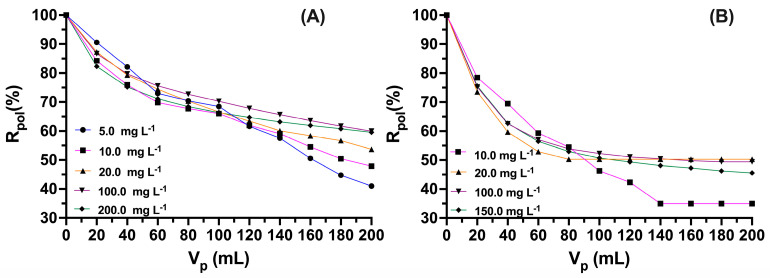
Retention profiles at different antibiotic concentrations: (**A**) poly(4-vinylbenzyl) trimethylammonium chloride (pH: 9.0, IS: 0.0 mol L^−1^) and (**B**) *N*-alkylated chitosan (pH: 7.0, IS: 0.0 mol L^−1^).

In the studies varying the concentration of nalidixic acid (see Figure 7), it can be observed that the retention percentages increase significantly with increasing concentration of nalidixic for the interaction with poly[(4-vinylbenzyl) trimethylammonium chloride], retaining almost 60% of the initial concentration of the antibiotic in the cell with a concentration of 200 mg L^−1^, that is, the polymer for this case removes approximately 140 mg L^−1^. Additionally, for a concentration of 100 mg L^−1^ approximately 70 mg L^−1^ is removed. N-alkylated chitosan removes 55% of the initial concentration (100 mg L^−1^) and 53.0% for 150 mg L^−1^. This decrease in the retention percentages between the two polymers under study is mainly based on the interactions between nalidixic and the polymeric chains that differ structurally [47,48]. On the other hand, it can be observed that at low concentrations of nalidixic, they present low removal percentages given the low availability of antibiotics in the solution, which causes an increase in the competition of the polymeric chains for the nalidixic molecules, increasing possible repulsions between polymer chains and actively available groups for maximum retention [49].

**Figure 8:**
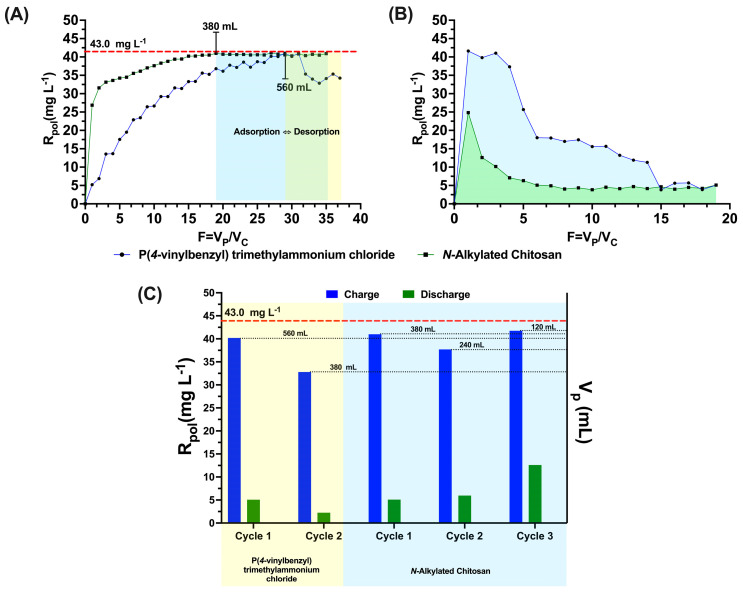
(**A**) Profile of maximum retention capacity (charge 1) and (**B**) discharge profile (discharge 1) and (**C**) summary of charge and discharge for the different polymers.

One of the important processes in the application of materials in the removal of contaminants is to know the maximum retention capacity or degree of saturation of the material. In our case, the maximum retention capacity is studied using the enrichment method. This method consists of having a concentration of analyte in the reservoir at established experimental conditions, which is passed to a continuous volume in the ultrafiltration cell that contains the polymer and the antibiotic under equally established conditions [14,50]. For this case, 20 mL tubes were collected, and several tubes were necessary to achieve maximum saturation of the polymer. Saturation studies were performed using a nalidixic acid concentration of 43.0 mg L^−1^, pH 9.0 and 0.0150 g for poly[(4-vinylbenzyl) trimethylammonium chloride] and pH 7.0 and 0.0291 g for *N*-alkylated chitosan. In Figure 8A, the concentration profile for both polymers can be observed as the degree of saturation of the polymer increases depending on the filtration factor. The results presented in this graph correlate with the percentages obtained in the previous results, clearly demonstrating that poly[(4-vinylbenzyl) trimethylammonium chloride] has a greater saturation capacity due to the functionality of the quaternary ammonium groups and the presence of aromatic rings that improve adsorption processes with respect to *N*-alkylated chitosan. This can be reflected mainly by the volume of the permeate reached in the saturation process. In the case of poly[(4-vinylbenzyl) trimethylammonium chloride], it reaches saturation at 560 mL of Vp, while for N-alkylated chitosan, a Vp of 380 mL can clearly be correlated with the mg of antibiotic retained per g of polymer through the following equation [15]:

$${Pol}_{sat}=\frac{\left[C_{o}\right]V_{P_{total}}}{{Pol}_{mass}}$$

where ${Pol}_{sat}$ is the saturation of the polymer obtained in the $V_{P_{total}}\;$ (volume of total permeate in liters; $C_{o}$ is the concentration of antibiotic in the reservoir as in the ultrafiltration cell; and ${Pol}_{mass}$ is the mass of the polymer used in the experimental design. Thus, ${Pol}_{sat}\;$ is 1605.33 mg antibiotic/g of polymer for poly[(4-vinylbenzyl) trimethylammonium chloride] and 561.51 mg antibiotic/g of polymer for N-alkylated chitosan.

Figure 8B shows the discharge profiles for the first charge of both polymers under study. It should be noted that these studies are carried out to determine the reuse capacity that the polymers can achieve as optimal and friendly processes for future scenarios in water treatment. For the discharge of antibiotics, the washing method described above was used, using an acidic pH between 2.0 and 3.0, where the lowest percentages of removal were presented and the greatest repulsions between the analyte and the adsorbent were presented. In the same way, the results obtained can be correlated with the interaction capacity that the polymers present with nalidixic acid and that is also correlated with the results obtained in previous studies. *N*-Alkylated chitosan presented greater desorption (see Figure 8B), demonstrating that the lower the area under the curve was, the greater the degree of desorption, and the greater the area under the curve was, the lower the degree of desorption.

To check the charge and discharge efficiency, at least two charge and discharge cycles were performed, as shown in Figure 8C. The saturation capacity of the polymer decreases as the load cycles increase, which is reflected in the decrease in $V_{P_{total}}$ in each load, and this is directly correlated with the discharge capacity because the washing in this case retains antibiotic residues interacting with the functional groups of the polymer, which reduces the active sites available for subsequent cycles of reuse of the material. From this, it can be said that even if the pH of the solution changes, short-range interactions between the polymers and the analyte occur under loading conditions [51,52].

### 3.3. Structural and Energetic Description of Polymer–Antibiotic Interactions

**Figure 9:**
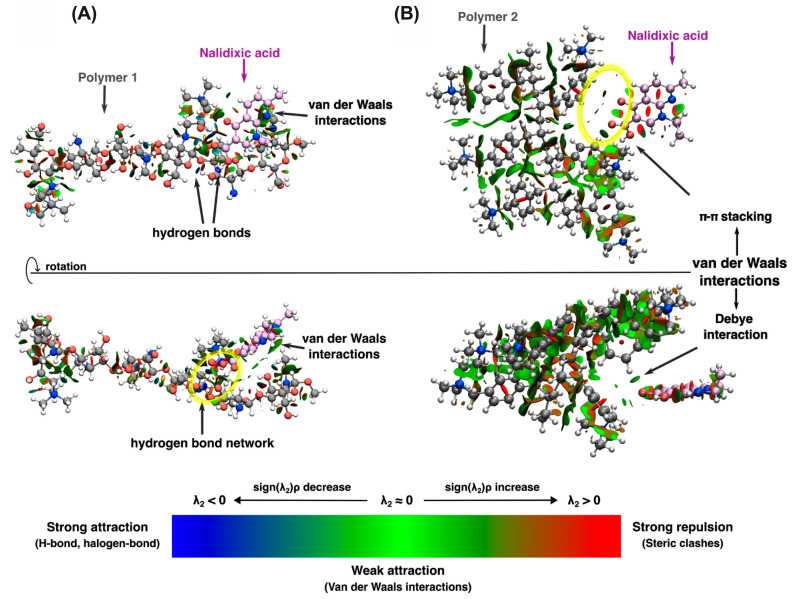
NCI plot isosurface (0.5 a.u.) of the noncovalent interaction for (**A**) I1-E2 and (**B**) I2-E2 interacting systems. Blue areas are strongly attractive, while green areas correspond to dispersive weak interactions.

The polymer–antibiotic interaction has been described in three aspects: (i) ∆E_int_, which provides quantitative information about the strength of the polymer–antibiotic interaction (see Table 1); (ii) the visual study of the noncovalent interaction via NCI analysis (see Figure 9); and (iii) the structural description of the interaction, which is a complement to NCI analysis.

The *polymer 1-antibiotic case, N-alkylated chitosan and nalidixic acid*: For this interaction, the Kick–Fukui method provides us with four potential interacting structures that have the following order in terms of ∆E_int_: I1-E2 < I1-E3 < I1-E4 < I1-E1; this means that the strongest stabilization is exhibited in the I1-E2 interacting system. Furthermore, this structure is also the most stable in terms of total electronic energies with respect to the other interacting systems. The next nearest stable structure was found at 11.30 kcal mol^−1^. When the NCI interactions were compared, it was found that all structures stabilized the polymer–antibiotic interaction by the formation of at least one H-bond between the carbonyl oxygen in nalidixic acid and the proton from the -OH groups in the polymer structure. Although these H-bonds were found in the four systems, the main difference between I1-E2 and the other systems is the number of H-bonds exhibited in the interacting region. For I1-E2, there are several H-bonds formed by the interaction of the carboxylic oxygens (both the carbonyl and alcohol oxygens) from the nalidixic molecule and some hydrogens and oxygens from the hydroxy and alkoxy groups in polymer structures, thus creating a hydrogen-bond network that confers stability and rigidity to the I1-E2 conformation. Structural evidence is depicted in NCI plots (see Figure 9 and Supplementary Material Figure S2).

*The polymer 2–antibiotic case, poly[(4-vinylbenzyl) trimethylammonium chloride] and nalidixic acid]*: For this interaction, the Kick–Fukui method provides us with three potential interacting structures that have the following order in terms of ∆E_int_: I2-E2 < I2-E3 < I2-E1. From this information, a particular case was noted. For the I2-E1 structure, the relaxation process started from an initial molecule with a high value of the coulombic integral of Fukui’s functions (between their fragments), but the optimized structure showed a system without interacting points. The low ∆E_relative_ (1.28 Kcal mol^−1^) with respect to the most stable structure could suggest a facility of adsorption and discharge of the antibiotic. Regarding those structures that exhibit interaction between polymer 2 and the antibiotic, the system with the highest interaction energy (∆E_int_) is also the lowest energy structure, and it is far from I2-E3 in 20.38 Kcal mol^−1^. The greatest concern from the results of Table 1 about the interaction energy on polymer 2–antibiotic systems is related to the close values of ∆E_int_ and the greater differences in the ∆E_relative_ for I2-E2 and I2-E3. These differences could be rationalized in terms of the kind of interactions that govern the polymer–antibiotic association and the structural arrangement of nalidixic acid over the polymer structure in both cases. NCI analysis showed that for these systems, the most important intramolecular interactions for conferring stabilization are van der Waals interactions. That is, although the polymer has methylated ammonium groups, these charged groups are not sufficient to electrostatically stabilize the interaction with nalidixic acid. In contrast, it is the van der Waals interactions that stabilize it, which are more intense when the contact area between the interacting fragments is larger. Something that must be taken into consideration is that van der Waals interactions are one of the most important forces in the adhesion of particles to any substrate. These forces originate due to the instantaneous dipole interactions between molecules formed due to the positions of the electrons surrounding the nuclei. Considering this, there are three types of dipole interactions, namely Keesom interactions (permanent dipole-permanent dipole), Debye interactions (permanent dipole-induced dipole), and London forces (dipole-induced dipole, as in π–π interactions) which in conjunction lead to van der Waals forces. Based on what has been mentioned before and the NCI plot (see Figure 8B), the stabilizing forces exhibited between polymer 2–antibiotic are mainly Debye and π–π stacking interactions.

The first important structural characteristic is the conformational structure of the polymer. In the I2-E2 system, the polymer conformation is more extended than in the I2-E3 system, in which the polymer is strongly coiled. For the first case (extended I2-E3 system), the antibiotic penetrates inside the polymer structure, exhibiting a larger interacting surface. Against this, in the I2-E3 system (coiled structure), only an interaction between the carboxylic oxygens of the antibiotic and the charged trimethylammonium group of polymer 2 was observed, which considerably reduced the interacting surface area between nalidixic acid and polymer 2, reducing the amount of weak stabilizing interactions, as can be observed in the NCI plots in Figure 9 (and Supplementary Material Figure S1).

## 4. Conclusions

The removal of nalidixic acid from two cationic polymers with different natures and structures was studied in this work. The spectroscopic findings verified the presence of major functional groups, including aromatic rings and quaternary ammonium groups. In pH studies, 75.0% removal rates were obtained for poly[(4-vinylbenzyl) trimethylammonium chloride] and 60.0% removal rates were obtained for N-alkylated chitosan. Given that nalidixic acid has a single pKa value of 5.95, the effect of pH on removal can be explained by the dissociation of carboxylate groups. As a result, for pH > 5.95, electrostatic interactions between the polymer and antibiotic are favored. Other kinds of interactions include those of hydrogen bonds or the interactions between the aromatic rings and the quinolone-type structure of an antibiotic, which influences the behavior of the elution curve. The maximum retention capacities for poly[(4-vinyl benzyl) trimethylammonium chloride] and *N*-alkylated chitosan were found to be approximately 1605.33 mg g^−1^ and 561.51 mg g^−1^, respectively. These effects demonstrated at the experimental level are reproduced by computational methods using the Kick–Fukui method. In conclusion, the technique coupled with these polymers makes it a promising method for the retention of antibiotics. In addition, it is a first step in its degradation.

## Figures and Tables

**Table 1:** Interaction energies (∆E_int_) for polymer 1 and polymer 2 with nalidixic acid calculated at the PM6//B3LYP/6-31g(d,p) level of theory using water as the solvent. Relative energies against the most stable interaction (∆E_relative_) are also reported. Total energies are in a.u., and ∆E_int_ and ∆E_relative_ are in Kcal mol^−1^.

**Polymer 1-Antibiotic**					
**Model**	**E_(polymer–antibiotic)_**	**E_(polymer)_**	**E_(antibiotic)_**	**∆E_int_**	**∆E_relative_**
I1-E1	−424.6326533	−4624.8794288	−799.7460253	−4.52 (−0.0071992)	16.01
I1-E2	−5424.6581731	−4624.8870078	−799.7414170	−18.67 (−0.0297483)	0.00
I1-E3	−5424.6310019	−4624.8758948	−799.7457499	−5.87 (−0.0093572)	17.05
I1-E4	−5424.6401586	−4624.8861272	−799.7458002	−5.17 (−0.0082312)	11.30
**Polymer 2–antibiotic**					
**Model**	**E_(polymer–antibiotic)_**	**E_(polymer)_**	**E_(antibiotic)_**	**∆E_int_**	**∆E_relative_**
I2-E1	−4251.8495288	−3452.1032797	−799.7460605	−0.12 (−0.0001886)	1.28
I2-E2	−4251.8515671	−3452.1035662	−799.7463146	−1.06 (−0.0016863)	0.00
I2-E3	−4251.8190859	−3452.0719998	−799.7457896	−0.81 (−0.0012965)	20.38

Solvation effects were introduced by the polarizable continuum model (PCM) using water as the solvent.

## Data Availability

Not applicable.

## Supplementary Materials

The following supporting information can be downloaded at https://www.mdpi.com/article/10.3390/polym15153185/s1, Figure S1: Study of antibiotic–membrane interaction at different pH. Table S1: Structures and Cartesian coordinates of minimum energy structures for N-alkylated chitosan (polymer 1), poly[(4-vinylbenzyl) trimethylammonium chloride] (polymer 2), and nalidixic acid (antibiotic) calculated at the PM6//B3LYP/6-31g(d,p) level of theory. Table S2: Chemical potential values obtained in the framework of the conceptual DFT for N-alkylated chitosan (polymer 1), poly[(4-vinylbenzyl) trimethylammonium chloride] (polymer 2), and nalidixic acid (antibiotic) calculated at the PM6//B3LYP/6-31g(d,p) level of theory. Table S3: Structures and Cartesian coordinates of minimum energy structures for the interaction between the N-alkylated chitosan (polymer 1) and the nalidixic acid (antibiotic) at PM6//B3LYP/6-31g(d,p) identified by the Kick–Fukui method. Table S4: Structures and Cartesian coordinates of minimum energy structures for the interaction between poly[(4-vinylbenzyl)trimethylammonium chloride] (polymer 2) and nalidixic acid (antibiotic) at PM6//B3LYP/6-31g(d,p) identified by the Kick–Fukui method. Table S5: Isosurface NCI plot (0.5 a.u.) of the noncovalent interaction for polymer and antibiotics.

## References

[1] González A., Kroll K.J., Silva-Sanchez C., Carriquiriborde P., Fernandino J.I., Denslow N.D., Somoza G.M. (2020). Steroid Hormones and Estrogenic Activity in the Wastewater Outfall and Receiving Waters of the Chascomús Chained Shallow Lakes System (Argentina). Sci. Total Environ..

[2] Muriuki C., Kairigo P., Home P., Ngumba E., Raude J., Gachanja A., Tuhkanen T. (2020). Mass Loading, Distribution, and Removal of Antibiotics and Antiretroviral Drugs in Selected Wastewater Treatment Plants in Kenya. Sci. Total Environ..

[3] Nzilu D.M., Madivoli E.S., Makhanu D.s., Otenda B.V., Kareru P.G., Kairigo P.k., Tuhkanen T. (2023). Environmental Remediation Using Nanomaterial as Adsorbents for Emerging Micropollutants. Environ. Nanotechnol. Monit. Manag..

[4] Rogowska J., Cieszynska-Semenowicz M., Ratajczyk W., Wolska L. (2020). Micropollutants in Treated Wastewater. Ambio.

[5] Jiang Z., Chen M., Lee X., Feng Q., Cheng N., Zhang X., Wang S., Wang B. (2023). Enhanced Removal of Sulfonamide Antibiotics from Water by Phosphogypsum Modified Biochar Composite. J. Environ. Sci..

[6] Li L., Zheng X., Chi Y., Wang Y., Sun X., Yue Q., Gao B., Xu S. (2020). Molecularly Imprinted Carbon Nanosheets Supported TiO_2_: Strong Selectivity and Synergic Adsorption-Photocatalysis for Antibiotics Removal. J. Hazard. Mater..

[7] Xu H., Wang B., Zhao R., Wang X., Pan C., Jiang Y., Zhang X., Ge B. (2022). Adsorption Behavior and Performance of Ammonium onto Sorghum Straw Biochar from Water. Sci. Rep..

[8] Xie Y., Kong F., Mi Z., Huang H., Xia C., Ma Z., Li S., Zhang Q., Meng Z. (2023). High-Efficiency Removal of Antibiotics through Self-Assembly Formation of Layered Double Hydroxides in Wastewater. J. Water Process Eng..

[9] Palacio D.A., Becerra Y., Urbano B.F., Rivas B.L. (2020). Antibiotics Removal Using a Chitosan-Based Polyelectrolyte in Conjunction with Ultrafiltration Membranes. Chemosphere.

[10] Fradj A.B., Hamouda S.B., Ouni H., Lafi R., Gzara L., Hafiane A. (2014). Removal of Methylene Blue from Aqueous Solutions by Poly(Acrylic Acid) and Poly(Ammonium Acrylate) Assisted Ultrafiltration. Sep. Purif. Technol..

[11] Dasgupta J., Sikder J., Mandal T., Adhikari U. (2015). Reactive Red 120 Retention through Ultrafiltration Enhanced by Synthetic and Natural Polyelectrolytes. J. Hazard. Mater..

[12] Palencia M., Martínez J.M., Arrieta Á. (2017). Removal of Acid Blue 129 Dye by Polymer-Enhanced Ultrafiltration (PEUF). J. Sci. Technol. Appl..

[13] Arce J.M., Palencia Luna V.J., Alemán Y.E., Lerma T.A., García-Quintero A. (2022). Removal of Synthetic Organic Species through Biomaterials Obtained from Fish Scales: A Case Study in a LRA System in the Municipality of San Pelayo—Córdoba. J. Sci. Technol. Appl..

[14] Oyarce E., Butter B., Santander P., Sánchez J. (2021). Polyelectrolytes Applied to Remove Methylene Blue and Methyl Orange Dyes from Water via Polymer-Enhanced Ultrafiltration. J. Environ. Chem. Eng..

[15] Palacio D.A., Urbano B.F., Rivas B.L. (2021). Water-Soluble Polymers with the Ability to Remove Amoxicillin as Emerging Pollutant from Water. Environ. Technol. Innov..

[16] Muñoz C., Palacio D.A., Rivas B.L., Muñoz C., Palacio D.A., Rivas B.L. (2020). Effect of Solvent Behavior of Nalidixic Acid by Ultraviolet Spectroscopy. J. Chil. Chem. Soc..

[17] Patiño Y., Pilehvar S., Díaz E., Ordóñez S., De Wael K. (2017). Electrochemical Reduction of Nalidixic Acid at Glassy Carbon Electrode Modified with Multi-Walled Carbon Nanotubes. J. Hazard. Mater..

[18] Zarei M., Beheshti Nahand F., Khataee A., Hasanzadeh A. (2019). Removal of Nalidixic Acid from Aqueous Solutions Using a Cathode Containing Three-Dimensional Graphene. J. Water Process Eng..

[19] Khataee A., Lotfi R., Hasanzadeh A., Iranifam M., Joo S.W. (2016). A Flow Injection Chemiluminescence Method for Determination of Nalidixic Acid Based on KMnO_4_–Morin Sensitized with CdS Quantum Dots. Spectrochim. Acta Part A Mol. Biomol. Spectrosc..

[20] Wu Q., Li Z., Hong H. (2013). Adsorption of the Quinolone Antibiotic Nalidixic Acid onto Montmorillonite and Kaolinite. Appl. Clay Sci..

[21] Rivas B.L., Pereira E.D., Palencia M., Sánchez J. (2011). Water-Soluble Functional Polymers in Conjunction with Membranes to Remove Pollutant Ions from Aqueous Solutions. Prog. Polym. Sci..

[22] Palencia M. (2015). Liquid-Phase Polymer-Based Retention: Theory, Modeling, and Application for the Removal of Pollutant Inorganic Ions. J. Chem..

[23] Stewart J.J. (2007). Optimization of Parameters for Semiempirical Methods V: Modification of NDDO Approximations and Application to 70 Elements. J. Mol. Model..

[24] Tirado-Rives J., Jorgensen W.L. (2008). Performance of B3LYP Density Functional Methods for a Large Set of Organic Molecules. J. Chem. Theory Comput..

[25] Rassolov V.A., Ratner M.A., Pople J.A., Redfern P.C., Curtiss L.A. (2001). 6-31G* Basis Set for Third-row Atoms. J. Comput. Chem..

[26] Frisch M., Trucks G., Schlegel H., Scuseria G., Robb M., Cheeseman J., Scalmani G., Barone V., Petersson G., Nakatsuji H. (2016). Gaussian 16.

[27] Yañez O., Báez-Grez R., Inostroza D., Pino-Rios R., Rabanal-León W.A., Contreras-García J., Cardenas C., Tiznado W. (2021). Kick–Fukui: A Fukui Function-Guided Method for Molecular Structure Prediction. J. Chem. Inf. Model..

[28] Yañez O., Garcia V., Garza J., Orellana W., Vásquez-Espinal A., Tiznado W. (2019). (Li6Si5) 2–5: The Smallest Cluster-Assembled Materials Based on Aromatic Si56− Rings. Chem. Eur. J..

[29] Osorio E., Ferraro M.B., Oña O.B., Cardenas C., Fuentealba P., Tiznado W. (2011). Assembling Small Silicon Clusters Using Criteria of Maximum Matching of the Fukui Functions. J. Chem. Theory Comput..

[30] Yañez O., Vásquez-Espinal A., Inostroza D., Ruiz L., Pino-Rios R., Tiznado W. (2017). A Fukui Function-guided Genetic Algorithm. Assessment on Structural Prediction of Sin (N = 12–20) Clusters. J. Comput. Chem..

[31] Grigoryan V.G., Springborg M. (2003). Structure and Energetics of Ni Clusters with up to 150 Atoms. Chem. Phys. Lett..

[32] Grigoryan V.G., Springborg M. (2004). Structural and Energetic Properties of Nickel Clusters: 2 ≤ N ≤ 150. Phys. Rev. B.

[33] Grigoryan V., Alamanova D., Springborg M. (2005). Structure and Energetics of Nickel, Copper, and Gold Clusters. Eur. Phys. J. At. Mol. Opt. Plasma Phys. Vol..

[34] Johnson E.R., Keinan S., Mori-Sánchez P., Contreras-García J., Cohen A.J., Yang W. (2010). Revealing Noncovalent Interactions. J. Am. Chem. Soc..

[35] Contreras-García J., Johnson E.R., Keinan S., Chaudret R., Piquemal J.-P., Beratan D.N., Yang W. (2011). NCIPLOT: A Program for Plotting Noncovalent Interaction Regions. J. Chem. Theory Comput..

[36] Pino-Rios R., Yañez O., Inostroza D., Ruiz L., Cardenas C., Fuentealba P., Tiznado W. (2017). Proposal of a Simple and Effective Local Reactivity Descriptor through a Topological Analysis of an Orbital-weighted Fukui Function. J. Comput. Chem..

[37] Lu T., Chen F. (2012). Multiwfn: A Multifunctional Wavefunction Analyzer. J. Comput. Chem..

[38] Sajomsang W., Gonil P., Saesoo S. (2009). Synthesis and Antibacterial Activity of Methylated N-(4-N,N-Dimethylaminocinnamyl) Chitosan Chloride. Eur. Polym. J..

[39] Jiang F., Deng Y., Yeh C.-K., Sun Y. (2014). Quaternized Chitosans Bind onto Preexisting Biofilms and Eradicate Pre-Attached Microorganisms. J. Mater. Chem. B.

[40] Palacio D.A., Vásquez V., Rivas B.L. (2020). Chromate Ion Removal by Water-Soluble Functionalized Chitosan. Polym. Adv. Technol..

[41] Zhao H., Liu X., Cao Z., Zhan Y., Shi X., Yang Y., Zhou J., Xu J. (2016). Adsorption Behavior and Mechanism of Chloramphenicols, Sulfonamides, and Non-Antibiotic Pharmaceuticals on Multi-Walled Carbon Nanotubes. J. Hazard. Mater..

[42] Pavez P., Toro-Labbé A., Encinas M.V. (2006). Photophysics and Photochemistry of Nalidixic Acid†. Photochem. Photobiol..

[43] Homayoonfal M., Mehrnia M.R. (2014). Amoxicillin Separation from Pharmaceutical Solution by PH Sensitive Nanofiltration Membranes. Sep. Purif. Technol..

[44] Villamizar-Sarmiento M.G., Molina-Soto E.F., Guerrero J., Shibue T., Nishide H., Moreno-Villoslada I., Oyarzun-Ampuero F.A. (2019). A New Methodology to Create Polymeric Nanocarriers Containing Hydrophilic Low Molecular-Weight Drugs: A Green Strategy Providing a Very High Drug Loading. Mol. Pharm..

[45] Malviya R., Kr Sharma P. (2014). Poly-Electrolyte Complex: A Novel System for Biomedical Applications and Recent Patents. Recent Pat. Nanotechnol..

[46] Solis F.J., de la Cruz M.O. (2000). Collapse of Flexible Polyelectrolytes in Multivalent Salt Solutions. J. Chem. Phys..

[47] Li Z., Liu Y., Zou S., Lu C., Bai H., Mu H., Duan J. (2020). Removal and Adsorption Mechanism of Tetracycline and Cefotaxime Contaminants in Water by NiFe2O4-COF-Chitosan-Terephthalaldehyde Nanocomposites Film. Chem. Eng. J..

[48] Zheng C., Zheng H., Hu C., Wang Y., Wang Y., Zhao C., Ding W., Sun Q. (2020). Structural Design of Magnetic Biosorbents for the Removal of Ciprofloxacin from Water. Bioresour. Technol..

[49] Jawad A.H., Abdulhameed A.S., Mastuli M.S. (2020). Acid-Factionalized Biomass Material for Methylene Blue Dye Removal: A Comprehensive Adsorption and Mechanism Study. J. Taibah Univ. Sci..

[50] Sánchez J., Espinosa C., Pooch F., Tenhu H., del Pizarro G.C., Oyarzún D.P. (2018). Poly(N,N-Dimethylaminoethyl Methacrylate) for Removing Chromium (VI) through Polymer-Enhanced Ultrafiltration Technique. React. Funct. Polym..

[51] Ahmed M.J. (2017). Adsorption of Quinolone, Tetracycline, and Penicillin Antibiotics from Aqueous Solution Using Activated Carbons: Review. Environ. Toxicol. Pharmacol..

[52] Yu B., Bai Y., Ming Z., Yang H., Chen L., Hu X., Feng S., Yang S.-T. (2017). Adsorption Behaviors of Tetracycline on Magnetic Graphene Oxide Sponge. Mater. Chem. Phys..

