# Supplementary material for: Comparative Study of the Removal Efficiency of Nalidixic Acid by Poly[(4-vinylbenzyl)trimethylammonium Chloride] and N-Alkylated Chitosan through the Ultrafiltration Technique and Its Approximation through Theoretical Calculations
Source: Polymers (Basel). 2023 Jul 27;15(15):3185. doi: 10.3390/polym15153185 (PMC10421493; doi:10.3390/polym15153185)

polymers

MDPI

Supplementary Information

Comparative Study of the Removal Efficiency of Nalidixic Acid by Poly[(4-vinylbenzyl)trimethylammonium Chloride] and N-Alkylated Chitosan through the Ultrafiltration Technique and Its Approximation through Theoretical Calculations

Daniel A. Palacio [1], Carla Muñoz [1], Manuel Meléndrez [2], Walter A. Rabanal-León [3], Juliana A. Murillo-López [4], Manuel Palencia [5] and Bernabé L. Rivas [1,*]

1 Departamento de Polímeros, Facultad de Ciencias Químicas, Universidad de Concepción, Edmundo Larenas 129, Casilla 160-C, Concepción 4070409, Chile; dapalacio@udec.cl (D.A.P.)
2 Departamento de Ingeniería de Materiales (DIMAT), Facultad de Ingeniería, Universidad de Concepción, Edmundo Larenas 270, Casilla 160-C, Concepción 4070409, Chile; mmelendrez@udec.cl
3 Laboratorio de Modelamiento Computacional en Sistemas Inorgánicos y Organometálicos (Lab-MCSIO), Departamento de Química Analítica e Inorgánica, Facultad de Ciencias Químicas, Universidad de Concepción, Edmundo Larenas 129, Casilla 160-C, Concepción 4070409, Chile
4 Departamento de Ciencias Químicas, Facultad de Ciencias Exactas, Universidad Andrés Bello, Autopista Concepción–Talcahuano 7100, Talcahuano 4260000, Chile
5 Departamento de Química, Facultad de Ciencias Naturales y Exactas, Grupo de Investigación en Ciencias con Aplicaciones Tecnológicas (GI-CAT), Universidad del Valle, Calle 13#100-00, Cali 25360, Colombia
* Correspondence: brivas@udec.cl

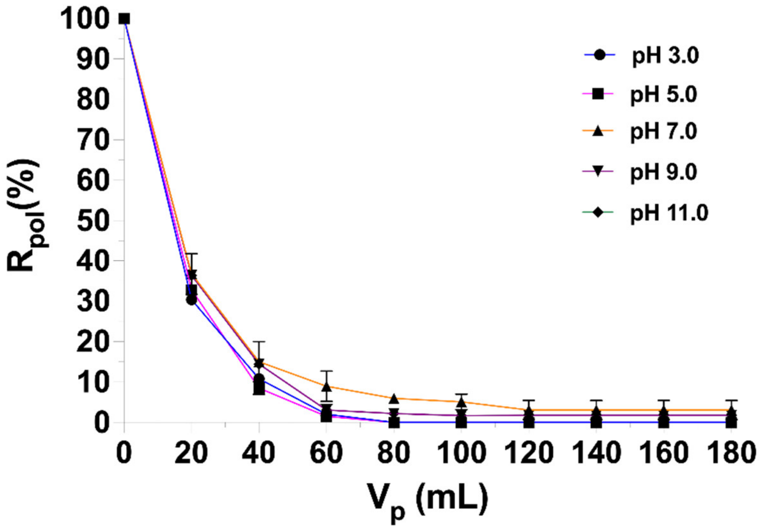

Figure S1. Study of antibiotic-membrane interaction at different pH.

Table S1. Structures and Cartesian coordinates of minimum energy structures for the N-alkylated chitosan (polymer-1), the poly(4-vinylbenzyl) trimethylammonium chloride (polymer-2), and nadilixic acid (antibiotic) calculated at PM6//B3LYP/6-31g(d,p) level of theory.

Polymer-1: alkylated chitosan (Charge 2 multiplicity 1)

C	-13.654469000	0.857365000	0.613960000
C	-12.715120000	1.158015000	-0.569186000
C	-11.024090000	-0.411369000	0.332819000
C	-12.002782000	-0.873471000	1.442721000
C	-13.467867000	-0.627273000	1.006387000
H	-12.928575000	0.478052000	-1.428642000
H	-13.349726000	1.492567000	1.486184000
H	-10.899266000	-1.093520000	-0.524574000
H	-11.815797000	-0.205029000	2.336744000
H	-14.176748000	-0.928526000	1.810712000
O	-9.803573000	-0.136547000	1.025400000
C	-8.563852000	-0.243659000	0.281791000
C	-8.624352000	0.512191000	-1.057779000
C	-7.569373000	0.402310000	1.272911000
H	-8.356934000	-1.323136000	0.108141000
C	-7.238366000	0.902404000	-1.636830000
H	-9.315468000	1.387023000	-0.976829000
H	-7.908002000	1.421011000	1.573157000
C	-6.231228000	1.348346000	-0.553322000
H	-6.813738000	-0.011779000	-2.148346000
H	-6.285152000	2.407275000	-0.241283000
O	-4.954553000	0.978983000	-1.076345000
C	-3.802481000	1.628188000	-0.476399000
C	-3.556618000	0.973216000	0.904800000
C	-2.686834000	1.298949000	-1.493635000
H	-3.977295000	2.724473000	-0.393588000
C	-2.129179000	1.277819000	1.414637000
H	-4.329527000	1.301127000	1.641763000
H	-2.732181000	0.224909000	-1.791432000
C	-1.107988000	0.834317000	0.332609000
H	-2.033425000	2.398650000	1.541835000
H	-1.066187000	-0.248860000	0.118985000
O	0.129435000	1.394957000	0.729244000
C	1.319998000	0.839337000	0.102936000
C	1.786768000	1.877638000	-0.934650000
C	2.314899000	0.703632000	1.269851000
H	1.104502000	-0.146553000	-0.367552000

H	-12.979723000	-5.563605000	0.044586000
N	-9.997275000	-4.611408000	-1.493716000
C	-7.303134000	-0.484083000	2.501067000
H	-8.139263000	-1.187226000	2.683199000
H	-6.351712000	-1.045028000	2.395320000
O	-7.091644000	0.324681000	3.662163000
H	-7.936435000	0.744329000	3.950042000
O	-9.171163000	-0.448545000	-1.986811000
H	-9.815233000	-0.010082000	-2.592569000
N	-7.316409000	1.998139000	-2.635504000
H	-7.783802000	1.674309000	-3.482864000
H	-7.860150000	2.786477000	-2.281986000
O	-3.663603000	-0.451638000	0.762099000
H	-4.566573000	-0.661285000	0.367649000
C	-2.712798000	2.230353000	-2.715021000
H	-3.645233000	2.091845000	-3.299907000
H	-1.833581000	2.063946000	-3.365963000
O	-2.756264000	3.605637000	-2.320695000
H	-1.971225000	3.816097000	-1.756923000
N	-1.836149000	0.651052000	2.712875000
H	-1.371928000	1.247489000	3.403353000
C	-2.061456000	-0.690431000	3.001327000
H	-2.543898000	-1.310991000	2.223967000
O	-1.728988000	-1.110463000	4.108397000
C	1.965428000	-0.465296000	2.205018000
H	0.985980000	-0.296512000	2.698119000
H	2.750601000	-0.618084000	2.969028000
O	1.774935000	-1.679202000	1.470462000
H	2.604730000	-1.905946000	0.982756000
O	1.097294000	1.637998000	-2.172535000
H	0.108114000	1.724961000	-2.026180000
N	3.844066000	2.995261000	-1.917134000
H	3.363326000	3.202236000	-2.805118000
C	3.803245000	4.196336000	-1.032299000
H	3.269067000	5.032929000	-1.537001000
H	3.260813000	4.017965000	-0.074361000
C	5.259980000	4.603268000	-0.719689000

H	15.969602000	-0.311022000	2.163565000
H	14.333134000	-0.938428000	1.911340000
H	15.747404000	-2.010205000	1.744987000
C	-15.107705000	1.165441000	0.270173000
H	-15.786920000	0.879531000	1.085467000
H	-15.260790000	2.242556000	0.099689000
H	-15.449618000	0.648216000	-0.635936000
C	14.839560000	-4.613441000	-1.451805000
H	13.881741000	-5.051901000	-1.746737000
H	15.147170000	-3.818075000	-2.136195000
H	15.616640000	-5.383770000	-1.369255000

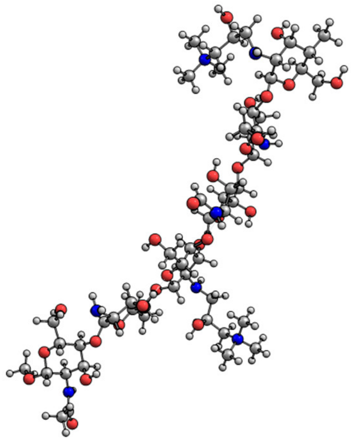

C	3.275409000	1.748517000	-1.339066000	H	5.955220000	4.224520000	-1.512609000
H	1.526045000	2.903816000	-0.579452000	C	5.475963000	6.131281000	-0.602612000
H	2.416175000	1.657845000	1.837041000	H	4.932498000	6.627779000	-1.447051000
C	4.213643000	1.277401000	-0.205669000	H	6.557011000	6.351355000	-0.802294000
H	3.324909000	0.969744000	-2.164152000	O	5.571087000	3.947868000	0.521864000
H	4.696526000	2.078832000	0.396952000	H	6.549142000	3.830867000	0.615436000
O	5.147244000	0.425565000	-0.864576000	N	5.092537000	6.806400000	0.710118000
C	6.445293000	0.267864000	-0.233270000	C	7.607521000	0.917990000	-2.433579000
C	6.356476000	-0.822797000	0.855946000	H	6.846921000	1.720263000	-2.383710000
C	7.326106000	-0.197989000	-1.414539000	H	8.621151000	1.347558000	-2.293889000
H	6.781363000	1.236286000	0.195914000	O	7.650893000	0.380580000	-3.759201000
C	7.742025000	-1.383570000	1.271210000	H	6.758415000	0.066206000	-4.035584000
H	5.649943000	-1.623042000	0.534699000	O	5.874029000	-0.237794000	2.073911000
H	6.885872000	-1.094273000	-1.910912000	H	4.900854000	-0.010229000	1.978026000
C	8.653600000	-1.640323000	0.049964000	N	7.639259000	-2.654582000	2.032463000
H	8.243758000	-0.619346000	1.932875000	H	7.192661000	-2.481643000	2.934709000
H	8.477258000	-2.588198000	-0.499123000	H	7.050994000	-3.330986000	1.543791000
O	9.986351000	-1.529531000	0.551259000	C	10.233368000	-4.639854000	0.300807000
C	10.993926000	-2.234808000	-0.218545000	H	9.360688000	-4.507475000	0.974109000
C	12.208023000	-1.275087000	-0.204203000	H	10.671554000	-5.646023000	0.444599000
C	11.284475000	-3.546877000	0.545449000	O	9.658236000	-4.544733000	-1.008773000
H	10.641430000	-2.430485000	-1.256077000	H	10.318954000	-4.826382000	-1.686549000
C	13.464225000	-2.040319000	-0.700643000	O	12.463318000	-0.846130000	1.140803000
H	12.010472000	-0.368993000	-0.820892000	H	11.627591000	-0.484947000	1.539210000
H	11.396709000	-3.340993000	1.636679000	N	14.638578000	-1.174391000	-0.867124000
C	13.686080000	-3.268013000	0.224219000	H	14.799893000	-0.866261000	-1.835862000
H	13.212336000	-2.439945000	-1.732187000	C	15.492209000	-0.639784000	0.098426000
H	13.835248000	-3.031073000	1.299245000	O	16.365518000	0.122530000	-0.330339000
O	-11.343136000	0.915198000	-0.164492000	C	-10.730393000	-5.771729000	-2.127353000
O	-6.286180000	0.502834000	0.625779000	H	-10.362906000	-5.978206000	-3.148689000
O	-1.397468000	1.566977000	-0.893341000	H	-10.612670000	-6.699809000	-1.539142000
O	3.594082000	0.332731000	0.715843000	H	-11.821469000	-5.567397000	-2.196864000
O	8.625249000	-0.532523000	-0.888698000	C	-8.533745000	-4.976828000	-1.353709000
O	12.519265000	-4.121764000	0.056372000	H	-8.083218000	-5.224829000	-2.333706000
O	14.771946000	-4.093450000	-0.098931000	H	-7.940625000	-4.150077000	-0.922310000
C	-12.685215000	2.622281000	-1.039465000	H	-8.392862000	-5.859324000	-0.701307000
H	-13.342191000	2.765955000	-1.918167000	C	-10.116574000	-3.399397000	-2.386302000
H	-11.648499000	2.924745000	-1.299585000	H	-9.621917000	-2.506681000	-1.948532000
O	-13.051504000	3.563214000	-0.023688000	H	-9.641482000	-3.572261000	-3.371290000
H	-14.003792000	3.443636000	0.225898000	H	-11.172367000	-3.138668000	-2.586373000
O	-13.714366000	-1.478173000	-0.132043000	C	3.722795000	6.383194000	1.182221000

H	-14.575880000	-1.243182000	-0.551142000	H	3.694031000	5.297938000	1.408362000
N	-11.605617000	-2.253340000	1.834564000	H	3.434387000	6.909803000	2.113060000
H	-11.562955000	-2.301363000	2.864013000	H	2.940442000	6.606334000	0.434304000
C	-12.427474000	-3.392931000	1.348699000	C	5.091828000	8.305550000	0.486674000
H	-13.525204000	-3.192160000	1.335672000	H	4.332039000	8.611371000	-0.257196000
H	-12.282895000	-4.247545000	2.049923000	H	4.865611000	8.855157000	1.420439000
C	-12.007734000	-3.797810000	-0.082286000	H	6.073116000	8.668292000	0.127329000
H	-12.173969000	-2.953422000	-0.798292000	C	6.105409000	6.475719000	1.783682000
C	-10.557825000	-4.324147000	-0.104392000	H	6.083701000	5.395150000	2.033960000
H	-10.483071000	-5.252395000	0.512575000	H	7.135234000	6.727229000	1.471232000
H	-9.904520000	-3.577202000	0.416134000	H	5.903429000	7.031301000	2.718515000
O	-12.912011000	-4.793356000	-0.567981000	C	15.374036000	-0.994756000	1.536598000

Polymer-2: (Charge 6 multiplicity 1)

C	-6.826581000	2.866375000	-0.079773000	C	2.755962000	4.272852000	0.590391000	H	11.425867000	1.880616000	0.176984000
H	-7.146486000	2.197504000	-0.887036000	H	1.382172000	4.802392000	2.184490000	H	11.549947000	2.377887000	1.880540000
H	-6.475248000	3.793678000	-0.546146000	H	4.018931000	3.414748000	-0.952204000	C	5.047480000	-7.787224000	-1.409378000
H	-7.718660000	3.113915000	0.507030000	C	2.141773000	-4.570990000	-0.548205000	H	4.900934000	-6.694554000	-1.513560000
C	-5.741261000	2.230623000	0.794583000	C	2.532856000	-5.918872000	-0.497601000	H	5.099371000	-8.017106000	-0.328737000
H	-5.411071000	2.997618000	1.543752000	C	1.424415000	-4.111706000	-1.665566000	H	6.038899000	-8.023859000	-1.841201000
C	-4.520367000	1.831825000	-0.057991000	C	2.205660000	-6.791504000	-1.544529000	C	3.936596000	-8.222927000	-3.565492000
H	-4.739862000	0.892331000	-0.602954000	H	3.083720000	-6.287910000	0.368724000	H	3.139958000	-8.767114000	-4.106050000
H	-4.356141000	2.582329000	-0.854996000	C	1.095283000	-4.982496000	-2.704058000	H	3.778173000	-7.140787000	-3.734195000
C	-3.234204000	1.662897000	0.775796000	H	1.131352000	-3.057272000	-1.720903000	H	4.896276000	-8.487463000	-4.049747000
H	-3.490272000	1.055601000	1.683960000	C	1.477272000	-6.326484000	-2.647478000	C	4.211818000	-10.045836000	-1.934205000
C	-2.178611000	0.886747000	-0.036609000	H	0.533298000	-4.615870000	-3.564908000	H	5.186964000	-10.334886000	-2.370959000
H	-1.745041000	1.550642000	-0.809821000	C	6.897808000	-1.528780000	0.757767000	H	4.231656000	-10.349005000	-0.870638000
H	-2.671418000	0.073612000	-0.605828000	C	6.213835000	-0.324769000	0.999745000	H	3.439382000	-10.656411000	-2.438665000
C	-1.053929000	0.293926000	0.837180000	C	8.188598000	-1.477628000	0.205405000	C	-4.974752000	-6.112175000	2.110972000
H	-0.699476000	1.093722000	1.540344000	C	6.810945000	0.901251000	0.709188000	H	-5.625958000	-6.448315000	1.283065000
C	0.140984000	-0.093898000	-0.059040000	H	5.206967000	-0.346186000	1.418954000	H	-3.964871000	-5.940230000	1.692099000
H	0.495524000	0.805956000	-0.599482000	C	8.784358000	-0.252539000	-0.095912000	H	-4.890280000	-6.955999000	2.822101000
H	-0.210487000	-0.796088000	-0.847654000	H	8.731441000	-2.403569000	0.008045000	C	-6.879590000	-5.187882000	3.368590000
C	1.318210000	-0.742043000	0.693381000	C	8.106038000	0.946508000	0.169163000	H	-6.824521000	-6.009897000	4.108056000
H	0.909964000	-1.628022000	1.261307000	H	9.779847000	-0.235621000	-0.543478000	H	-7.315164000	-4.314080000	3.888282000
C	2.357572000	-1.273904000	-0.314305000	H	-9.198471000	-2.492541000	-1.289170000	H	-7.598963000	-5.502782000	2.589378000
H	2.989449000	-0.435679000	-0.680603000	H	-4.613462000	-4.708467000	-0.554398000	H	-3.923416000	-1.830245000	2.597091000
H	1.841771000	-1.660021000	-1.220806000	H	1.199128000	-7.001192000	-3.457279000	C	-11.018876000	-0.299195000	-2.330770000
C	3.255164000	-2.383287000	0.273037000	C	8.733889000	2.270951000	-0.160377000	H	-11.336969000	-1.340704000	-2.522191000
H	3.670057000	-2.006221000	1.245808000	H	9.344929000	2.194080000	-1.094741000	H	-11.490676000	0.333124000	-3.107097000
C	4.436045000	-2.655208000	-0.678106000	H	7.943396000	3.029467000	-0.392130000	H	-9.925365000	-0.248568000	-2.493872000

H	4.880738000	-1.692887000	-1.001349000	C	3.020694000	5.697138000	0.192419000	C	-12.902232000	0.085330000	-0.790296000
H	4.059376000	-3.125535000	-1.608241000	H	3.178388000	5.772686000	-0.913256000	H	-13.417194000	0.709843000	-1.544953000
C	5.534543000	-3.543678000	-0.060801000	H	2.122574000	6.331385000	0.400686000	H	-13.277882000	-0.947776000	-0.914879000
H	5.047799000	-4.457911000	0.367425000	C	-1.563048000	6.771043000	-3.013531000	H	-13.232158000	0.439877000	0.204462000
C	-6.296787000	1.032355000	1.604428000	H	-2.478305000	6.873847000	-3.649432000	C	-10.948964000	1.587529000	-0.748462000
H	-5.450727000	0.503630000	2.095302000	H	-0.788672000	6.328257000	-3.690250000	H	-11.198516000	1.962587000	0.261201000
H	-6.908404000	1.426868000	2.443105000	C	2.589141000	-8.245893000	-1.455569000	H	-9.855546000	1.693948000	-0.884290000
C	-2.684982000	3.017388000	1.289577000	H	2.606881000	-8.577055000	-0.385963000	H	-11.432920000	2.266473000	-1.476267000
H	-1.774984000	2.822266000	1.897462000	H	1.805078000	-8.882880000	-1.938420000	C	-4.587099000	-4.460959000	3.899189000
H	-3.419361000	3.447743000	2.004421000	C	-5.681698000	-3.735826000	1.769648000	H	-4.939184000	-3.545602000	4.409788000
C	-1.555363000	-0.872193000	1.720074000	H	-6.146052000	-2.856589000	2.285440000	H	-4.500490000	-5.251243000	4.669249000
H	-0.674726000	-1.387834000	2.173123000	H	-6.431605000	-4.045992000	0.997771000	H	-3.564549000	-4.262131000	3.525082000
H	-2.096625000	-0.433118000	2.586763000	C	-10.764912000	-0.750706000	0.120141000	H	6.262677000	1.825217000	0.902603000
C	1.953753000	0.191028000	1.748262000	H	-11.103239000	-0.410119000	1.131829000				
H	1.305971000	0.183934000	2.652194000	H	-11.185274000	-1.782424000	0.008718000				
H	2.913334000	-0.256425000	2.093962000	N	9.624894000	2.818464000	0.964645000				
C	2.451780000	-3.662436000	0.606633000	N	-1.099682000	8.172597000	-2.592439000				
H	1.491829000	-3.363505000	1.092247000	N	4.229898000	6.319121000	0.905239000				
H	3.001463000	-4.219505000	1.395804000	N	3.946444000	-8.563628000	-2.093386000				
C	6.506862000	-4.004003000	-1.167873000	N	-11.397297000	0.157366000	-0.941039000				
H	7.062305000	-3.134277000	-1.569809000	N	-5.518858000	-4.873643000	2.783787000				
H	5.923782000	-4.399044000	-2.024405000	C	7.486780000	-5.071694000	-0.690462000				
C	6.265031000	-2.843860000	1.109405000	H	8.187947000	-4.678354000	0.055860000				
H	5.544095000	-2.708397000	1.944481000	H	6.974241000	-5.924053000	-0.229931000				
H	7.035579000	-3.536777000	1.512134000	H	8.084441000	-5.461447000	-1.523077000				
C	-7.101919000	0.061987000	0.788845000	C	-2.171404000	8.869265000	-1.786829000				
C	-8.505156000	0.115655000	0.812916000	H	-3.125375000	8.934116000	-2.342371000				
C	-6.467559000	-0.916508000	0.006226000	H	-2.372466000	8.342895000	-0.834528000				
C	-9.260375000	-0.791661000	0.058964000	H	-1.876665000	9.904084000	-1.526920000				
H	-9.002911000	0.866146000	1.428535000	C	0.164191000	8.085560000	-1.769834000				
C	-7.222988000	-1.823561000	-0.738941000	H	-0.007781000	7.543067000	-0.821025000				
H	-5.376396000	-0.966182000	-0.020542000	H	0.971030000	7.559072000	-2.312612000				
C	-8.619228000	-1.769719000	-0.714392000	H	0.549531000	9.088283000	-1.504587000				
H	-6.721419000	-2.580260000	-1.345098000	C	-0.824143000	8.979016000	-3.843809000				
C	-2.380050000	4.012313000	0.207038000	H	-0.494453000	10.007554000	-3.602101000				
C	-1.101381000	4.074426000	-0.371456000	H	-0.027433000	8.522547000	-4.460732000				
C	-3.373602000	4.899155000	-0.243257000	H	-1.724666000	9.068779000	-4.479812000				
C	-0.830036000	4.981537000	-1.396538000	C	4.043403000	6.277289000	2.404152000				
H	-0.311904000	3.405919000	-0.020888000	H	3.113719000	6.788535000	2.715196000				
C	-3.106908000	5.801634000	-1.272303000	H	3.996889000	5.237409000	2.779970000				
H	-4.367427000	4.875096000	0.206993000	H	4.880149000	6.774002000	2.931462000				

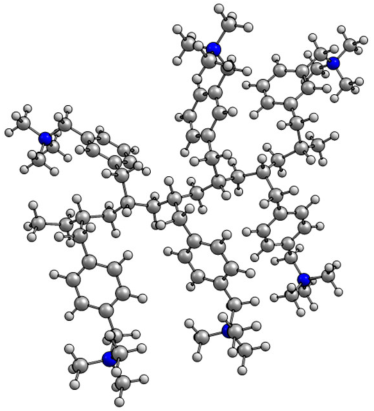

C	-1.832158000	5.849209000	-1.857575000	C	4.349800000	7.762021000	0.462506000
H	0.165924000	5.003960000	-1.843400000	H	5.214941000	8.263467000	0.936579000
H	-3.901144000	6.461876000	-1.625672000	H	4.485551000	7.844546000	-0.632361000
C	-2.408655000	-1.914205000	1.056149000	H	3.449899000	8.348469000	0.727761000
C	-3.604723000	-2.316462000	1.673814000	C	5.497398000	5.582863000	0.539992000
C	-2.018649000	-2.537183000	-0.140964000	H	5.456124000	4.522489000	0.852180000
C	-4.389398000	-3.330742000	1.108839000	H	5.684708000	5.604543000	-0.549533000
C	-2.811126000	-3.533085000	-0.712161000	H	6.383367000	6.027023000	1.032736000
H	-1.090900000	-2.227432000	-0.633839000	C	10.045972000	4.225510000	0.597003000
C	-3.999028000	-3.934986000	-0.093560000	H	10.598940000	4.250815000	-0.360723000
H	-2.503311000	-4.003131000	-1.648002000	H	9.174774000	4.900070000	0.495824000
C	2.208662000	1.604425000	1.311197000	H	10.709047000	4.666618000	1.365512000
C	1.601111000	2.669135000	1.999169000	C	8.874472000	2.856297000	2.275408000
C	3.080324000	1.894284000	0.248643000	H	7.958714000	3.471040000	2.204807000
C	1.869939000	3.990269000	1.641446000	H	8.572224000	1.842917000	2.602271000
H	0.916835000	2.467203000	2.824592000	H	9.493345000	3.285729000	3.085903000
C	3.348554000	3.213385000	-0.114148000	C	10.859067000	1.961204000	1.122812000
H	3.543128000	1.069841000	-0.306910000	H	10.604674000	0.935725000	1.450924000

Antibiotic: nadilixic acid (Charge 0 multiplicity 1)

C	1.884922000	-0.136539000	-0.058099000
C	1.082128000	-1.364604000	0.084817000
C	-0.388410000	-1.117593000	0.024403000
C	-0.951229000	0.175794000	-0.150728000
C	1.250978000	1.091688000	-0.231756000
H	-0.890074000	-3.200730000	0.271199000
C	-1.301373000	-2.187763000	0.139146000
H	1.853055000	2.005003000	-0.341067000
C	-3.129849000	-0.609539000	-0.092812000
C	-2.672592000	-1.944009000	0.080837000
H	-3.398161000	-2.766904000	0.166424000
N	-2.271572000	0.419980000	-0.206730000
N	-0.091415000	1.278734000	-0.283861000
C	-0.646132000	2.646386000	-0.428481000
H	-1.551809000	2.560951000	-1.056986000
H	0.107789000	3.236875000	-0.984733000
C	-0.980075000	3.300219000	0.913235000
H	-1.372456000	4.322554000	0.737627000
H	-1.755603000	2.722782000	1.454581000
H	-0.082113000	3.383345000	1.558970000
O	1.530287000	-2.512513000	0.239740000
C	3.370052000	-0.173726000	-0.024254000

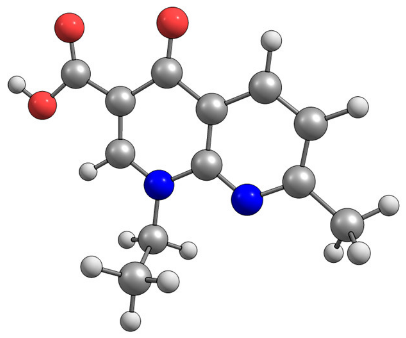

O	4.066921000	-1.165483000	0.144354000
O	3.925176000	1.063642000	-0.204556000
H	4.897886000	0.931024000	-0.158897000
C	-4.601306000	-0.282740000	-0.133215000
H	-5.213315000	-1.167924000	-0.392136000
H	-4.937346000	0.080502000	0.862871000
H	-4.798716000	0.530907000	-0.858583000

Table S2. Chemical potential values obtained in the framework of the conceptual-DFT for the N-alkylated chitosan (polymer-1), the poly(4-vinylbenzyl) trimethylammonium chloride (polymer-2), and nadilixic acid (antibiotic) calculated at PM6//B3LYP/6-31g(d,p) level of theory.

System	Property	Value (a.u.)	Value (eV)	FF-assingment*
antibiotic	μ (chemical potential)	-0.214514	-5.8372	f^+
Polymer-1	μ (chemical potential)	-0.172988	-4.7072	f^-
Polymer-2	μ (chemical potential)	-0.172468	-4.6931	f^-

* FF: Fukui's function, f^+: electrophilic Fukui's function, f^-: nucleophilic Fukui's function.

Table S3. Structures and Cartesian coordinates of minimum energy structures for the interaction between the N-alkylated chitosan (polymer-1) and the nalidixic acid (antibiotic) at PM6//B3LYP/6-31g(d,p) identified by Kick-Fukui method.

**Structure 1: I1-E1 (220 atoms)**											
C	-8.071074000	-0.503210000	4.074649000	H	-5.441042000	1.639902000	-3.005746000	H	-3.529439000	-5.949387000	-3.219537000
C	-8.236613000	-0.058391000	2.696688000	C	-7.090325000	2.390622000	-1.068369000	H	-5.183765000	-5.708400000	-2.635281000
C	-8.377682000	-1.142664000	1.708068000	H	-6.640594000	1.690931000	0.939952000	O	-4.230011000	-3.527756000	-0.958324000
C	-8.449157000	-2.498148000	2.100504000	H	-6.960404000	3.269374000	-1.730178000	H	-5.203215000	-3.357550000	-0.922289000
C	-8.147245000	-1.849820000	4.409878000	O	-8.363595000	2.358488000	-0.426219000	N	-3.821515000	-6.399052000	-1.117004000
H	-8.366278000	0.187197000	-0.017335000	C	-9.484629000	2.911457000	-1.157086000	C	-6.273586000	-0.351280000	-3.423470000
C	-8.450634000	-0.860237000	0.322098000	C	-10.656606000	2.035149000	-0.645017000	H	-5.587290000	-1.213681000	-3.337485000
H	-8.047567000	-2.163267000	5.471453000	C	-9.629027000	4.384980000	-0.711936000	H	-7.320313000	-0.685710000	-3.266280000
C	-8.698638000	-3.230097000	-0.113304000	H	-9.333453000	2.814013000	-2.253479000	O	-6.275504000	0.128650000	-4.771416000
C	-8.611442000	-1.890299000	-0.583100000	C	-12.002943000	2.700426000	-1.036671000	H	-5.361352000	0.357570000	-5.060685000
H	-8.674047000	-1.693512000	-1.653921000	H	-10.583575000	0.998766000	-1.046562000	O	-4.481129000	0.845843000	1.023204000
N	-8.614445000	-3.530573000	1.209452000	H	-9.499428000	4.463680000	0.394206000	H	-3.567167000	0.436353000	0.969566000
N	-8.351755000	-2.836762000	3.484358000	C	-12.039496000	4.128553000	-0.429481000	N	-5.803297000	3.618014000	0.600919000
C	-8.382696000	-4.274663000	3.926023000	H	-11.989224000	2.816677000	-2.164790000	H	-5.294466000	3.569775000	1.484385000
H	-9.063258000	-4.844046000	3.247100000	H	-11.949188000	4.176614000	0.676640000	H	-5.205153000	4.127030000	-0.051668000
H	-8.839396000	-4.324953000	4.942293000	O	12.596318000	-1.119785000	0.193392000	C	-8.684552000	5.351874000	-1.442470000
C	-6.983764000	-4.867977000	3.918808000	O	7.564702000	-0.355469000	0.427437000	H	-7.700584000	5.413898000	-0.934300000
H	-7.011264000	-5.923957000	4.227332000	O	2.822009000	-1.152534000	-1.596646000	H	-9.134682000	6.361854000	-1.509108000
H	-6.524819000	-4.840223000	2.920649000	O	-2.254860000	0.061502000	-0.274648000	O	-8.349943000	4.884389000	-2.755100000
H	-6.301882000	-4.352372000	4.607688000	O	-7.158599000	1.193265000	-1.885967000	H	-9.134643000	4.959151000	-3.349848000
O	-8.276283000	1.122278000	2.353589000	O	-10.950552000	4.865662000	-1.052943000	O	-10.600265000	1.958598000	0.784150000
C	-7.833390000	0.474872000	5.125247000	O	-13.182387000	4.889193000	-0.713785000	H	-9.666471000	1.704947000	1.083615000
O	-7.416217000	1.616647000	5.079037000	C	13.845692000	-2.926424000	-0.609854000	N	-13.171455000	1.868585000	-0.719620000
O	-8.135055000	-0.052749000	6.366171000	H	14.535457000	-3.098897000	-1.457673000	H	-13.534864000	1.330857000	-1.518282000
H	-7.953927000	0.587075000	7.113213000	H	12.803241000	-3.130941000	-0.934725000	C	-13.779843000	1.627958000	0.512257000
C	-8.875339000	-4.362446000	-1.057575000	O	14.075683000	-3.926452000	0.389287000	O	-14.711011000	0.815115000	0.494482000
H	-9.769314000	-4.230259000	-1.690460000	H	15.020437000	-3.897201000	0.691799000	C	12.744709000	5.736609000	-1.382204000
H	-8.014226000	-4.451621000	-1.743356000	O	15.146700000	1.038785000	0.560879000	H	12.477203000	6.056877000	-2.405156000
H	-8.979889000	-5.338984000	-0.556543000	H	16.015649000	0.736852000	0.203967000	H	12.678723000	6.625373000	-0.728705000
C	14.817537000	-1.309932000	1.187891000	N	12.940105000	1.918791000	2.375783000	H	13.811528000	5.422678000	-1.395897000
C	13.971999000	-1.484386000	-0.087679000	H	12.801131000	1.917020000	3.397836000	C	10.424728000	5.122758000	-0.843196000
C	12.353756000	0.216051000	0.711039000	C	13.907443000	2.996092000	2.038311000	H	10.078834000	5.461899000	-1.838352000
C	13.246488000	0.529470000	1.939121000	H	14.977824000	2.686910000	2.100751000	H	9.721285000	4.341498000	-0.502942000
C	14.720429000	0.168928000	1.630295000	H	13.785623000	3.817947000	2.781793000	H	10.320060000	5.982040000	-0.154070000
H	14.331326000	-0.806032000	-0.898069000	C	13.653212000	3.526044000	0.609126000	C	11.929126000	3.459991000	-1.860957000
H	14.381970000	-1.949997000	1.999025000	H	13.806227000	2.716525000	-0.148740000	H	11.298402000	2.603677000	-1.538707000
H	12.393599000	0.937260000	-0.122165000	C	12.261642000	4.184170000	0.508367000	H	11.579278000	3.748917000	-2.870755000

H	12.910163000	-0.163259000	2.768344000	H	12.211969000	5.069535000	1.187527000	H	12.965025000	3.091651000	-1.978676000
H	15.371926000	0.374085000	2.509886000	H	11.504764000	3.463410000	0.913477000	C	-2.473733000	-6.079104000	-0.518000000
O	11.042826000	0.042932000	1.253522000	O	14.680877000	4.460815000	0.270367000	H	-2.438836000	-5.036737000	-0.141976000
C	9.906124000	0.283172000	0.385278000	H	14.757276000	5.185650000	0.935244000	H	-2.246820000	-6.736948000	0.343796000
C	10.069476000	-0.387485000	-0.990377000	N	11.852140000	4.617965000	-0.895279000	H	-1.655016000	-6.216782000	-1.247229000
C	8.768366000	-0.349574000	1.218703000	C	8.401369000	0.494028000	2.451327000	C	-3.837611000	-7.855785000	-1.535046000
H	9.794203000	1.384567000	0.266799000	H	9.236754000	1.152875000	2.757949000	H	-3.044446000	-8.081131000	-2.272664000
C	8.736984000	-0.662683000	-1.737264000	H	7.489214000	1.099243000	2.268711000	H	-3.674124000	-8.527322000	-0.670531000
H	10.704271000	-1.304640000	-0.901523000	O	8.030964000	-0.354577000	3.542193000	H	-4.804700000	-8.142728000	-1.989308000
H	9.020501000	-1.397439000	1.504616000	H	8.823197000	-0.816343000	3.905676000	C	-4.890551000	-6.183029000	-0.069274000
C	7.607728000	-1.137040000	-0.796266000	O	10.765445000	0.603446000	-1.776356000	H	-4.872874000	-5.140740000	0.310201000
H	8.406099000	0.306447000	-2.214525000	H	11.356255000	0.168335000	-2.435852000	H	-5.902613000	-6.378762000	-0.467944000
H	7.603227000	-2.212142000	-0.540454000	N	8.871696000	-1.684964000	-2.804899000	H	-4.747699000	-6.850407000	0.801257000
O	6.402895000	-0.699560000	-1.425044000	H	9.427783000	-1.321571000	-3.579285000	C	-13.354900000	2.331576000	1.750377000
C	5.179299000	-1.349111000	-0.989010000	H	9.348867000	-2.520698000	-2.464524000	H	-13.758602000	1.834453000	2.647458000
C	4.834118000	-0.824713000	0.426096000	O	4.982107000	0.603208000	0.439095000	H	-12.254482000	2.363139000	1.873821000
C	4.166717000	-0.875125000	-2.055993000	H	5.922257000	0.826875000	0.153758000	H	-13.721582000	3.370138000	1.781497000
H	5.307967000	-2.455038000	-1.003529000	C	4.267586000	-1.680056000	-3.360693000	C	16.266711000	-1.726664000	0.960532000
C	3.362146000	-1.134526000	0.781786000	H	5.249512000	-1.521758000	-3.851768000	H	16.883397000	-1.553505000	1.853604000
H	5.533577000	-1.248516000	1.186803000	H	3.451707000	-1.415304000	-4.059879000	H	16.344962000	-2.800763000	0.730087000
H	4.269453000	0.220868000	-2.235515000	O	4.233972000	-3.088420000	-3.106717000	H	16.738456000	-1.187096000	0.128731000
C	2.449713000	-0.543508000	-0.326445000	H	3.402393000	-3.320573000	-2.623953000	C	-13.549385000	5.052782000	-2.107990000
H	3.228183000	-2.258720000	0.777716000	N	2.974343000	-0.640453000	2.111945000	H	-12.685271000	5.366624000	-2.700778000
H	2.454416000	0.556946000	-0.424427000	H	2.424865000	-1.286067000	2.686031000	H	-13.982824000	4.122808000	-2.485995000
O	1.169393000	-1.099571000	-0.092621000	C	3.218989000	0.649888000	2.569143000	H	-14.303413000	5.848615000	-2.060642000
C	0.049384000	-0.444818000	-0.751441000	H	3.795156000	1.329080000	1.913996000				
C	-0.358739000	-1.363201000	-1.918743000	O	2.798454000	0.960085000	3.682430000				
C	-1.031363000	-0.388665000	0.343137000	C	-0.737387000	0.682459000	1.405413000				
H	0.328654000	0.573407000	-1.104879000	H	0.190305000	0.440715000	1.964307000				
C	-1.810967000	-1.161186000	-2.417880000	H	-1.582928000	0.792764000	2.110403000				
H	-0.144722000	-2.425711000	-1.647870000	O	-0.453916000	1.949710000	0.803080000				
H	-1.194236000	-1.387222000	0.811159000	H	-1.224114000	2.237549000	0.254088000				
C	-2.822932000	-0.797283000	-1.307562000	O	0.427068000	-1.022894000	-3.072704000				
H	-1.789813000	-0.296400000	-3.152918000	H	1.401100000	-1.138676000	-2.859249000				
H	-3.334861000	-1.648946000	-0.808203000	N	-2.341096000	-2.328550000	-3.171337000				
O	-3.722850000	0.104545000	-1.944064000	H	-1.793934000	-2.443333000	-4.037674000				
C	-5.000923000	0.329522000	-1.287629000	C	-2.376263000	-3.620477000	-2.425271000				
C	-4.818661000	1.448286000	-0.238064000	H	-1.821418000	-4.406800000	-2.984919000				
C	-5.899927000	0.784072000	-2.457254000	H	-1.896230000	-3.560882000	-1.420224000				
H	-5.363462000	-0.607506000	-0.812833000	C	-3.856323000	-4.026209000	-2.254610000				
C	-6.098386000	2.248244000	0.108166000	H	-4.493913000	-3.531242000	-3.031604000				

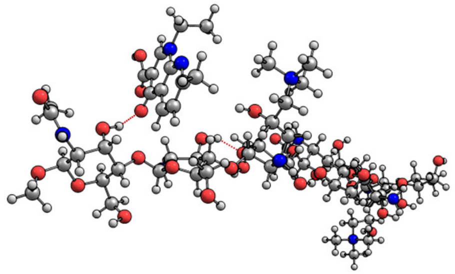

H	-3.977896000	2.111463000	-0.553088000	C	-4.111337000	-5.549447000	-2.349640000				
**Structure 2: I1-E2 (220 atoms)**											
C	6.707781000	0.977318000	2.007193000	C	11.380095000	-1.150030000	-1.005236000	O	11.365935000	-5.312084000	0.407890000
C	6.988057000	2.112939000	1.136230000	H	9.281290000	-1.437615000	-1.683151000	H	12.193929000	-4.916896000	0.037262000
C	8.092932000	2.975995000	1.588471000	H	10.324455000	-2.454779000	1.974914000	O	9.360795000	-0.524057000	0.167066000
C	8.816225000	2.700179000	2.770726000	C	12.128888000	-1.221515000	0.352876000	H	8.527057000	-0.889567000	0.575857000
C	7.451127000	0.743689000	3.158401000	H	11.796876000	-1.976281000	-1.661772000	N	11.565092000	0.101921000	-1.750536000
H	7.913869000	4.362124000	-0.068821000	H	11.740275000	-0.552614000	1.149510000	H	11.725986000	-0.029192000	-2.758396000
C	8.462219000	4.127838000	0.851258000	O	-12.235780000	1.978653000	0.178777000	C	11.454474000	1.419542000	-1.309631000
H	7.215653000	-0.132411000	3.800159000	O	-7.410516000	0.389583000	0.329477000	O	11.578676000	2.286541000	-2.181870000
C	10.182732000	4.587179000	2.493137000	O	-2.696655000	0.186455000	-1.894021000	C	-13.705683000	-4.859743000	-0.715983000
C	9.497497000	4.927177000	1.293675000	O	2.040668000	-1.885806000	-0.377613000	H	-13.562442000	-5.301708000	-1.718345000
H	9.794940000	5.820306000	0.740290000	O	7.316793000	-2.581190000	-1.427234000	H	-13.761328000	-5.692426000	0.008352000
N	9.848559000	3.486935000	3.218968000	O	12.040396000	-2.612363000	0.782306000	H	-14.697928000	-4.357306000	-0.709500000
N	8.486452000	1.547108000	3.547997000	O	13.503360000	-0.957016000	0.320389000	C	-11.284862000	-4.638395000	-0.323487000
C	9.204177000	1.259370000	4.839492000	C	-13.184123000	3.912795000	-0.736832000	H	-11.074633000	-5.144969000	-1.284808000
H	10.257772000	1.622187000	4.755933000	H	-13.897303000	4.160556000	-1.545533000	H	-10.433447000	-3.963648000	-0.118650000
H	9.263373000	0.153591000	4.973842000	H	-12.151992000	3.890035000	-1.146582000	H	-11.284074000	-5.419291000	0.460111000
C	8.491627000	1.920835000	6.006822000	O	-13.137222000	4.999750000	0.195332000	C	-12.516396000	-2.822752000	-1.434055000
H	9.031952000	1.728200000	6.946209000	H	-14.041889000	5.179357000	0.557352000	H	-11.733399000	-2.067826000	-1.206628000
H	8.430033000	3.012268000	5.894090000	O	-15.093645000	0.344519000	0.810227000	H	-12.262758000	-3.254490000	-2.421494000
H	7.467825000	1.549068000	6.145473000	H	-15.924412000	0.748789000	0.464670000	H	-13.476779000	-2.289488000	-1.559088000
O	6.378054000	2.347870000	0.093268000	N	-13.003698000	-0.740284000	2.655823000	C	3.044775000	4.510679000	-2.892653000
C	5.620125000	0.081639000	1.659432000	H	-12.812315000	-0.667949000	3.666851000	H	2.303224000	3.812916000	-2.458336000
O	4.708101000	0.256931000	0.856002000	C	-14.161658000	-1.652877000	2.464777000	H	2.670251000	5.532447000	-2.683534000
O	5.666701000	-1.109855000	2.317595000	H	-15.156125000	-1.151851000	2.536211000	H	3.040346000	4.387984000	-3.990768000
H	4.896901000	-1.764609000	2.006400000	H	-14.145155000	-2.410603000	3.282388000	C	5.335755000	5.391735000	-2.820315000
C	11.298325000	5.424606000	3.000494000	C	-14.084939000	-2.349843000	1.087896000	H	5.407736000	5.383003000	-3.923902000
H	12.125477000	5.481638000	2.271309000	H	-14.124774000	-1.598193000	0.259044000	H	4.973099000	6.395604000	-2.527905000
H	10.972783000	6.463353000	3.185507000	C	-12.849950000	-3.270251000	1.003290000	H	6.360928000	5.290140000	-2.419453000
H	11.731937000	5.055516000	3.944477000	H	-12.936980000	-4.090404000	1.757197000	C	4.334121000	4.402714000	-0.801104000
C	-14.347666000	2.645565000	1.214377000	H	-11.949912000	-2.680542000	1.315099000	H	3.601449000	3.673264000	-0.394346000
C	-13.536859000	2.560662000	-0.092531000	O	-15.285834000	-3.098499000	0.880877000	H	5.309183000	4.174148000	-0.322374000
C	-12.207472000	0.673497000	0.816085000	H	-15.453077000	-3.740123000	1.611425000	H	4.023635000	5.411338000	-0.477967000
C	-13.085444000	0.635157000	2.093194000	N	-12.600562000	-3.888977000	-0.368568000	C	11.234936000	1.752520000	0.122828000
C	-14.487689000	1.216138000	1.787555000	C	-8.275355000	-0.121281000	2.451981000	H	10.940698000	2.808044000	0.239727000
H	-14.043897000	1.895837000	-0.831937000	H	-9.201471000	-0.555451000	2.876242000	H	10.438696000	1.141012000	0.589577000
H	-13.777236000	3.266399000	1.953846000	H	-7.523270000	-0.920600000	2.287871000	H	12.152065000	1.617439000	0.719868000
H	-12.410708000	-0.101334000	0.058036000	O	-7.655680000	0.732981000	3.418013000	C	-15.710751000	3.289425000	0.983922000
H	-12.599714000	1.331847000	2.840915000	H	-8.310344000	1.374930000	3.781885000	H	-16.318972000	3.290254000	1.899246000
H	-15.131002000	1.203040000	2.696368000	O	-10.843047000	-0.243399000	-1.651363000	H	-15.612411000	4.340917000	0.673501000

O	-10.863206000	0.657403000	1.302309000
C	-9.826342000	0.140051000	0.430018000
C	-9.941826000	0.681645000	-1.006143000
C	-8.560583000	0.653863000	1.154471000
H	-9.905654000	-0.970723000	0.431352000
C	-8.620407000	0.628752000	-1.819025000
H	-10.398711000	1.702679000	-1.001570000
H	-8.627640000	1.750856000	1.341856000
C	-7.389450000	1.027351000	-0.975800000
H	-8.473946000	-0.435947000	-2.166604000
H	-7.212456000	2.110588000	-0.848873000
O	-6.293089000	0.338983000	-1.576845000
C	-4.968233000	0.853252000	-1.275914000
C	-4.637652000	0.519022000	0.199303000
C	-4.087707000	0.067887000	-2.275102000
H	-4.935359000	1.949666000	-1.469858000
C	-3.119555000	0.650682000	0.461340000
H	-5.226733000	1.164964000	0.893492000
H	-4.368403000	-1.011798000	-2.279193000
C	-2.373411000	-0.261748000	-0.547725000
H	-2.816812000	1.721713000	0.256010000
H	-2.567927000	-1.346351000	-0.453984000
O	-1.004294000	0.086716000	-0.441607000
C	-0.064003000	-0.873800000	-0.997634000
C	0.621560000	-0.138808000	-2.166168000
C	0.898869000	-1.184079000	0.164642000
H	-0.586399000	-1.794053000	-1.348977000
C	1.956168000	-0.795841000	-2.593500000
H	0.748365000	0.946563000	-1.922074000
H	1.247563000	-0.248909000	0.662208000
C	2.819505000	-1.127875000	-1.358454000
H	1.712430000	-1.765289000	-3.122398000
H	3.301843000	-0.262236000	-0.840945000
O	3.736983000	-2.126260000	-1.792341000
C	4.978167000	-2.189603000	-1.045840000
C	4.881430000	-3.346778000	-0.037143000
C	6.051352000	-2.494168000	-2.106703000
H	5.168964000	-1.215938000	-0.533178000
C	6.250027000	-3.745511000	0.567063000
H	4.341448000	-4.212968000	-0.488395000
H	5.845001000	-3.452811000	-2.636447000
H	-11.365053000	0.214802000	-2.352145000
N	-8.635975000	1.510084000	-3.012902000
H	-9.279230000	1.147121000	-3.716688000
H	-8.953691000	2.451392000	-2.778653000
O	-4.990860000	-0.847439000	0.460473000
H	-5.964764000	-0.976070000	0.233411000
C	-4.121913000	0.668629000	-3.688791000
H	-5.127897000	0.560173000	-4.143120000
H	-3.362085000	0.197568000	-4.341071000
O	-3.911103000	2.084269000	-3.661767000
H	-3.038723000	2.285029000	-3.242343000
N	-2.741942000	0.333990000	1.847495000
H	-2.055009000	0.960247000	2.277059000
C	-3.157570000	-0.803261000	2.531011000
H	-3.881802000	-1.472124000	2.029490000
O	-2.714685000	-0.994122000	3.662485000
C	0.293488000	-2.157544000	1.188915000
H	-0.444257000	-1.637637000	1.835164000
H	1.079596000	-2.619040000	1.815890000
O	-0.480823000	-3.179730000	0.553505000
H	0.111708000	-3.782930000	0.042617000
O	-0.207362000	-0.244309000	-3.333470000
H	-1.125610000	0.095365000	-3.118803000
N	2.765158000	0.010679000	-3.549056000
H	2.314073000	-0.052386000	-4.477997000
C	2.949636000	1.455980000	-3.219171000
H	3.020598000	2.014758000	-4.181546000
H	2.084563000	1.894182000	-2.659489000
C	4.232950000	1.665631000	-2.392533000
H	4.950860000	0.807207000	-2.547970000
C	5.019255000	2.957977000	-2.719519000
H	5.236824000	2.976656000	-3.815946000
H	6.020181000	2.869998000	-2.213366000
O	3.805052000	1.661018000	-1.031396000
H	4.611839000	1.609870000	-0.398163000
N	4.418954000	4.298053000	-2.305913000
C	6.255760000	-1.347667000	-3.115304000
H	6.038635000	-0.347465000	-2.679066000
H	7.283851000	-1.358027000	-3.527387000
O	5.409232000	-1.568460000	-4.242090000
H	4.455426000	-1.413061000	-3.970506000
H	-16.294625000	2.781829000	0.204859000
C	14.303767000	-1.692276000	-0.641405000
H	14.087968000	-2.762991000	-0.587832000
H	14.129864000	-1.294146000	-1.645225000
H	15.327533000	-1.480515000	-0.308624000

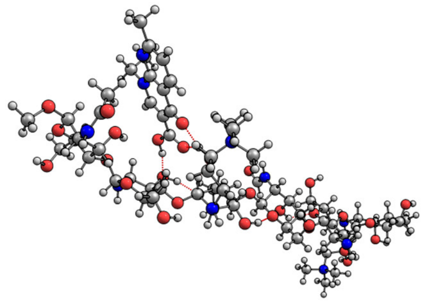

C	7.454308000	-3.581639000	-0.392315000	O	4.117863000	-2.932659000	1.120469000				
H	6.437553000	-3.063201000	1.458024000	H	3.228595000	-2.540015000	0.815687000				
H	7.818524000	-4.509683000	-0.875243000	N	6.284952000	-5.145436000	1.056398000				
O	8.452071000	-2.989050000	0.450638000	H	5.670131000	-5.252390000	1.864803000				
C	9.786766000	-2.903769000	-0.111731000	H	5.951263000	-5.797522000	0.344757000				
C	9.891754000	-1.495957000	-0.750095000	C	10.918890000	-4.527504000	1.520051000				
C	10.707922000	-3.059730000	1.120106000	H	9.964549000	-5.005552000	1.817779000				
H	9.958706000	-3.712078000	-0.856426000	H	11.656745000	-4.610269000	2.341000000				
**Structure 3: I1-E3 (220 atoms)**											
C	1.742460000	3.481263000	-1.819204000	C	13.211051000	-1.633784000	-0.888397000	O	10.051472000	-1.454895000	-4.189163000
C	3.149344000	3.557595000	-1.435360000	H	11.470729000	-1.106997000	0.381689000	H	10.828035000	-0.892536000	-4.424771000
C	3.638739000	4.918076000	-1.142073000	H	11.195677000	-3.980597000	-2.244311000	O	11.777271000	-3.152422000	0.317551000
C	2.802599000	6.050318000	-1.253693000	C	13.568861000	-2.763407000	-1.892142000	H	10.845138000	-3.413498000	0.541674000
C	0.967522000	4.629897000	-1.933969000	H	13.170691000	-0.664110000	-1.475846000	N	14.195367000	-1.429918000	0.182375000
H	5.650473000	4.269626000	-0.649550000	H	13.549101000	-3.792865000	-1.475643000	H	14.408841000	-0.441682000	0.374519000
C	4.978470000	5.135018000	-0.738278000	O	-11.979432000	-0.302688000	-0.872901000	C	14.791330000	-2.363699000	1.031407000
H	-0.094017000	4.551925000	-2.253993000	O	-6.806186000	0.104263000	0.921454000	O	15.527359000	-1.890774000	1.904371000
C	4.518064000	7.507455000	-0.593277000	O	-1.716262000	0.555210000	0.294619000	C	-13.423137000	-4.457694000	-3.047942000
C	5.419181000	6.415179000	-0.465518000	O	2.959572000	-1.683577000	0.346181000	H	-13.134084000	-4.518870000	-4.112656000
H	6.448152000	6.602499000	-0.154187000	O	8.260015000	-0.740701000	-0.601385000	H	-13.791915000	-5.455279000	-2.747316000
N	3.228213000	7.328010000	-0.983602000	O	12.622899000	-2.648699000	-2.992099000	H	-14.284825000	-3.759016000	-2.971891000
N	1.441465000	5.881896000	-1.654269000	O	14.826819000	-2.665442000	-2.503975000	C	-11.145044000	-5.009736000	-2.298742000
C	0.544749000	7.074620000	-1.851027000	C	-12.069253000	1.872272000	-1.878272000	H	-10.812145000	-5.120887000	-3.348744000
H	0.855520000	7.881504000	-1.143438000	H	-12.804244000	2.499994000	-2.418655000	H	-10.260229000	-4.710777000	-1.706725000
H	-0.494395000	6.790128000	-1.561711000	H	-11.470983000	1.288219000	-2.614936000	H	-11.452338000	-6.013335000	-1.948215000
C	0.604624000	7.557822000	-3.291108000	O	-11.085631000	2.720931000	-1.275528000	C	-11.778927000	-2.661372000	-2.665198000
H	-0.024753000	8.451284000	-3.423918000	H	-11.506630000	3.342463000	-0.636044000	H	-10.834847000	-2.358819000	-2.173739000
H	1.622513000	7.837578000	-3.596918000	O	-14.745466000	-0.216692000	0.810249000	H	-11.581041000	-2.670135000	-3.754368000
H	0.245513000	6.805876000	-4.006075000	H	-15.407918000	0.496473000	0.645456000	H	-12.521114000	-1.856627000	-2.478093000
O	3.892328000	2.583080000	-1.361316000	N	-12.893863000	-2.165094000	1.839284000	C	4.143947000	0.780009000	6.363566000
C	1.142041000	2.186695000	-2.087178000	H	-13.109382000	-2.238269000	2.848167000	H	3.670514000	0.071905000	5.655548000
O	1.553501000	1.051583000	-1.936808000	C	-14.044746000	-2.753092000	1.098481000	H	3.848541000	0.452996000	7.380467000
O	-0.138798000	2.329957000	-2.603155000	H	-14.996943000	-2.179906000	1.236768000	H	3.700126000	1.780158000	6.210286000
H	-0.546238000	1.457685000	-2.866813000	H	-14.227944000	-3.758961000	1.547674000	C	6.242387000	1.669256000	7.290619000
C	4.955229000	8.897755000	-0.306196000	C	-13.794298000	-2.880952000	-0.416191000	H	5.858975000	2.705821000	7.245852000
H	5.325934000	9.000235000	0.728780000	H	-13.567608000	-1.883776000	-0.878669000	H	6.002945000	1.280867000	8.299396000
H	5.784358000	9.203645000	-0.968017000	C	-12.705162000	-3.932713000	-0.718721000	H	7.345321000	1.718380000	7.216987000
H	4.154939000	9.645318000	-0.433113000	H	-13.059492000	-4.943749000	-0.399899000	C	6.172225000	-0.609645000	6.369340000
C	-13.006074000	1.485505000	0.512983000	H	-11.813787000	-3.721032000	-0.075071000	H	5.716551000	-1.298921000	5.628454000
C	-12.778258000	0.925175000	-0.897606000	O	-15.022062000	-3.235613000	-1.056517000	H	7.269681000	-0.655922000	6.246447000
C	-11.496345000	-0.807721000	0.364253000	H	-15.423908000	-4.048158000	-0.667083000	H	5.941638000	-1.015270000	7.373025000

C	-12.490480000	-0.765656000	1.553525000	N	-12.271679000	-4.003874000	-2.180462000	C	14.578383000	-3.825165000	0.867956000
C	-13.561866000	0.346179000	1.402715000	C	-8.195640000	-0.671013000	2.651742000	H	14.940311000	-4.379689000	1.749281000
H	-13.738154000	0.562599000	-1.346387000	H	-9.142908000	-1.239857000	2.719188000	H	13.511090000	-4.095009000	0.744542000
H	-12.015731000	1.806754000	0.939443000	H	-7.332350000	-1.368062000	2.670756000	H	15.125895000	-4.230131000	0.001889000
H	-11.137605000	-1.824533000	0.089943000	O	-8.020282000	0.113646000	3.835813000	C	-13.951214000	2.680836000	0.487925000
H	-11.865871000	-0.445423000	2.451728000	H	-8.819032000	0.667708000	4.004422000	H	-14.153788000	3.054996000	1.501574000
H	-13.852813000	0.728869000	2.409110000	O	-9.493968000	-0.173444000	-2.101625000	H	-13.534680000	3.525184000	-0.076585000
O	-10.416200000	0.031752000	0.806193000	H	-10.495881000	-0.071103000	-2.038454000	H	-14.921327000	2.444633000	0.030628000
C	-9.101770000	-0.264261000	0.282549000	N	-7.159206000	1.700006000	-2.450599000	C	15.144080000	-1.427377000	-3.190649000
C	-8.888264000	0.544093000	-1.013611000	H	-7.654307000	1.468594000	-3.313907000	H	14.328063000	-1.132986000	-3.857188000
C	-8.162420000	0.226468000	1.404976000	H	-7.528264000	2.599482000	-2.138672000	H	15.366924000	-0.647290000	-2.457567000
H	-8.995364000	-1.355094000	0.091128000	O	-4.130406000	-1.930304000	-0.161320000	H	16.040663000	-1.697520000	-3.762568000
C	-7.401497000	0.641626000	-1.437023000	H	-4.848978000	-1.642224000	-0.794973000				
H	-9.380801000	1.547330000	-0.923794000	C	-2.833086000	2.353842000	-0.725993000				
H	-8.365028000	1.290734000	1.665694000	H	-3.621766000	2.673122000	-1.437795000				
C	-6.473678000	0.915137000	-0.230802000	H	-1.833236000	2.636801000	-1.115795000				
H	-7.104153000	-0.347673000	-1.893220000	O	-3.119803000	3.111414000	0.454152000				
H	-6.376406000	1.970739000	0.084494000	H	-2.422861000	2.949981000	1.133598000				
O	-5.213165000	0.365036000	-0.626933000	N	-2.549309000	-2.621169000	2.111373000				
C	-4.151871000	0.441280000	0.360815000	H	-2.261235000	-2.736509000	3.086404000				
C	-4.056087000	-0.995604000	0.928095000	C	-2.700302000	-3.738779000	1.299778000				
C	-2.911931000	0.842097000	-0.466037000	H	-2.991606000	-3.577197000	0.245781000				
H	-4.380819000	1.188819000	1.151561000	O	-2.516341000	-4.848761000	1.797573000				
C	-2.704233000	-1.235171000	1.640798000	C	1.050336000	-3.027619000	0.030895000				
H	-4.919701000	-1.203399000	1.606667000	H	0.248613000	-3.565045000	0.577270000				
H	-2.877395000	0.264475000	-1.420353000	H	1.835756000	-3.743923000	-0.278131000				
C	-1.547496000	-0.834659000	0.687548000	O	0.376825000	-2.520181000	-1.128171000				
H	-2.660746000	-0.555292000	2.543885000	H	1.033108000	-2.153601000	-1.767063000				
H	-1.405518000	-1.467500000	-0.211750000	O	0.917854000	1.799475000	1.348797000				
O	-0.392054000	-0.764335000	1.510550000	H	-0.038941000	1.649140000	1.119510000				
C	0.860182000	-0.580187000	0.798150000	N	3.840656000	1.653388000	1.880834000				
C	1.570823000	0.540294000	1.576914000	H	3.454282000	2.604230000	1.784797000				
C	1.641719000	-1.902682000	0.895857000	C	3.962151000	1.311137000	3.328174000				
H	0.681613000	-0.282994000	-0.264803000	H	3.747782000	2.212054000	3.945981000				
C	3.017156000	0.728029000	1.061243000	H	3.238589000	0.529286000	3.662813000				
H	1.526053000	0.357916000	2.677295000	C	5.399919000	0.810123000	3.580498000				
H	1.751362000	-2.237504000	1.954139000	H	6.069103000	1.074432000	2.706102000				
C	3.762269000	-0.618504000	0.936505000	C	6.066345000	1.372387000	4.855861000				
H	2.962420000	1.193084000	0.018870000	H	5.911684000	2.482825000	4.865397000				
H	4.221338000	-1.012238000	1.868805000	H	7.176221000	1.247675000	4.759064000				
O	4.698336000	-0.407732000	-0.117029000	O	5.282266000	-0.621072000	3.634369000				

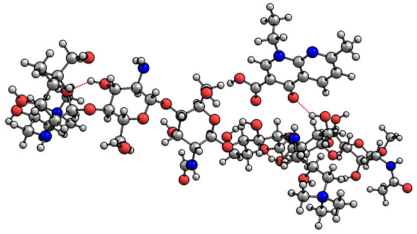

C	5.955137000	-1.115748000	-0.016092000	H	6.170801000	-1.050083000	3.566097000				
C	5.885388000	-2.363364000	-0.918962000	N	5.644431000	0.797397000	6.204021000				
C	6.970134000	-0.101324000	-0.583490000	C	7.151061000	1.146260000	0.298751000				
H	6.172771000	-1.392992000	1.037991000	H	6.756939000	1.007278000	1.331158000				
C	7.286931000	-2.990124000	-1.136416000	H	8.213466000	1.454215000	0.346239000				
H	5.371488000	-2.122343000	-1.878143000	O	6.483354000	2.251476000	-0.310831000				
H	6.692586000	0.200522000	-1.621846000	H	5.488313000	2.079771000	-0.338962000				
C	8.361837000	-1.919699000	-1.447019000	O	5.145632000	-3.401831000	-0.260961000				
H	7.583072000	-3.523124000	-0.186715000	H	4.221318000	-3.072200000	-0.047108000				
H	8.427930000	-1.589684000	-2.504413000	N	7.310002000	-3.981495000	-2.241757000				
O	9.580712000	-2.489063000	-0.968519000	H	6.718759000	-4.779240000	-2.001089000				
C	10.790790000	-1.936365000	-1.547765000	H	6.924868000	-3.585973000	-3.100830000				
C	11.775144000	-1.885203000	-0.354817000	C	10.477969000	-2.802597000	-3.954079000				
C	11.250650000	-2.935973000	-2.633225000	H	9.524916000	-3.370272000	-3.916165000				
H	10.605521000	-0.923013000	-1.969019000	H	11.091836000	-3.154761000	-4.805278000				
**Structure 4: I1-E4 (220 atoms)**											
C	-4.604200000	-2.721688000	3.580427000	C	-12.293244000	-1.803970000	-2.175980000	O	-8.622482000	-3.900575000	-0.437435000
C	-5.372320000	-1.489973000	3.484390000	H	-10.944711000	-0.041391000	-2.126079000	H	-9.430499000	-4.046152000	0.111818000
C	-5.379140000	-0.664618000	4.703836000	H	-9.695452000	-3.418615000	-3.613378000	O	-10.899778000	-0.989117000	-3.966682000
C	-4.648208000	-1.034402000	5.855055000	C	-12.261240000	-3.218847000	-2.815797000	H	-10.009163000	-0.640264000	-4.235213000
C	-3.884426000	-3.032158000	4.729313000	H	-12.288581000	-1.944763000	-1.050405000	N	-13.490492000	-1.013574000	-2.489676000
H	-6.706315000	0.843517000	3.882483000	H	-12.157333000	-3.237141000	-3.921806000	H	-13.882910000	-0.506293000	-1.684669000
C	-6.128786000	0.536410000	4.767746000	O	12.423517000	-0.078658000	-0.015186000	C	-14.095426000	-0.770225000	-3.723549000
H	-3.282336000	-3.965570000	4.769544000	O	7.205264000	1.148283000	-0.968510000	O	-15.056806000	0.005885000	-3.697915000
C	-5.365198000	0.858777000	7.041324000	O	2.369698000	2.038825000	0.717574000	C	10.765152000	-5.705821000	2.094936000
C	-6.127026000	1.294300000	5.919792000	O	-2.404070000	0.955311000	-1.536132000	H	10.348759000	-5.746711000	3.117850000
H	-6.700347000	2.220978000	5.987826000	O	-7.643466000	-0.085993000	-1.083928000	H	10.586871000	-6.690836000	1.627197000
N	-4.636427000	-0.287602000	7.008664000	O	-11.147666000	-3.923810000	-2.197810000	H	11.866759000	-5.581969000	2.184955000
N	-3.871568000	-2.232286000	5.839331000	O	-13.372390000	-4.034227000	-2.562125000	C	8.675072000	-4.846405000	1.108395000
C	-3.104568000	-2.668402000	7.058531000	C	12.322082000	2.174819000	-0.888094000	H	8.166067000	-4.973616000	2.082761000
H	-2.786947000	-1.761578000	7.628753000	H	11.546525000	2.273858000	-0.095904000	H	8.168888000	-4.014702000	0.583259000
H	-2.165757000	-3.171778000	6.726692000	H	11.816272000	2.101063000	-1.871339000	H	8.489640000	-5.767733000	0.524134000
C	-3.950164000	-3.589140000	7.921926000	O	13.080897000	3.382725000	-0.981971000	C	10.333548000	-3.283692000	2.037582000
H	-3.397700000	-3.882595000	8.827701000	H	13.540003000	3.573709000	-0.131484000	H	9.975963000	-2.404919000	1.461758000
H	-4.880121000	-3.109736000	8.257454000	O	14.518604000	-1.735953000	-0.908298000	H	9.764517000	-3.286144000	2.988477000
H	-4.228874000	-4.517896000	7.406587000	H	14.166273000	-1.411050000	-0.041020000	H	11.391148000	-3.106325000	2.308666000
O	-5.967408000	-1.128013000	2.466585000	N	11.991537000	-2.832977000	-2.283440000	C	-3.642982000	7.222737000	0.327339000
C	-4.583327000	-3.656487000	2.463982000	H	12.015919000	-3.058485000	-3.290470000	H	-3.199344000	6.469495000	-0.354361000
O	-5.371891000	-3.841490000	1.556841000	C	12.782166000	-3.862618000	-1.555629000	H	-3.370030000	8.216791000	-0.078303000
O	-3.455300000	-4.451967000	2.516303000	H	13.878078000	-3.642827000	-1.503958000	H	-3.153830000	7.136298000	1.314080000
H	-3.427793000	-5.139051000	1.790201000	H	12.694833000	-4.824651000	-2.112535000	C	-5.707070000	8.182153000	1.265557000

C	-5.340739000	1.647762000	8.298651000	C	12.266370000	-4.038452000	-0.109972000	H	-5.277277000	8.191182000	2.284904000
H	-4.994981000	2.681322000	8.123005000	H	12.405424000	-3.102593000	0.484296000	H	-5.493380000	9.171512000	0.817371000
H	-6.348459000	1.726301000	8.743947000	C	10.801819000	-4.521817000	-0.094222000	H	-6.806216000	8.105534000	1.367686000
H	-4.684319000	1.219866000	9.073958000	H	10.732413000	-5.532110000	-0.567842000	C	-5.734527000	7.144441000	-0.964572000
C	14.020357000	0.481036000	-1.836080000	H	10.198468000	-3.851844000	-0.760979000	H	-5.291126000	6.384640000	-1.639658000
C	13.240060000	0.973910000	-0.596595000	O	13.114596000	-4.957474000	0.580021000	H	-6.829291000	6.991732000	-0.955095000
C	11.606762000	-0.863138000	-0.885425000	H	13.219227000	-5.807858000	0.087922000	H	-5.547793000	8.133489000	-1.425344000
C	12.376492000	-1.409331000	-2.108227000	N	10.156073000	-4.582295000	1.286974000	C	-13.631434000	-1.431229000	-4.970895000
C	13.884085000	-1.052706000	-1.993109000	C	8.111110000	0.263068000	-2.949110000	H	-14.066942000	-0.948550000	-5.861248000
H	13.919973000	1.192551000	0.263029000	H	8.866037000	-0.499373000	-3.225482000	H	-12.531879000	-1.392181000	-5.098233000
H	13.587282000	0.955343000	-2.756370000	H	7.103999000	-0.200368000	-2.902300000	H	-13.932461000	-2.490264000	-5.015573000
H	11.176911000	-1.619856000	-0.196580000	O	7.989651000	1.226106000	-3.999511000	C	15.487757000	0.889855000	-1.740764000
H	11.981408000	-0.863366000	-3.021139000	H	8.874106000	1.591944000	-4.238951000	H	16.027444000	0.666686000	-2.670101000
H	14.448883000	-1.428490000	-2.877464000	O	10.140703000	-0.219413000	1.546967000	H	15.595431000	1.966720000	-1.556438000
O	10.604619000	0.006816000	-1.433407000	H	11.122435000	-0.048730000	1.610796000	H	16.007305000	0.360772000	-0.930872000
C	9.351894000	0.069842000	-0.713301000	N	8.341184000	1.986670000	2.502987000	C	-13.749926000	-4.243600000	-1.176864000
C	9.543512000	0.741826000	0.661347000	H	8.820693000	1.494272000	3.258869000	H	-12.880673000	-4.530524000	-0.578047000
C	8.474656000	0.954473000	-1.624766000	H	8.914565000	2.800096000	2.275553000	H	-14.230813000	-3.342592000	-0.786325000
H	8.943279000	-0.959672000	-0.601640000	O	4.622682000	0.546856000	-1.410996000	H	-14.466257000	-5.071639000	-1.251371000
C	8.190791000	1.087579000	1.332046000	H	5.503430000	0.186785000	-1.074332000				
H	10.220910000	1.630304000	0.568080000	C	3.645713000	2.184927000	2.670212000				
H	8.942372000	1.950235000	-1.800364000	H	4.557455000	1.881682000	3.223613000				
C	7.208508000	1.764618000	0.347890000	H	2.742869000	1.868488000	3.226318000				
H	7.722746000	0.123165000	1.689682000	O	3.713425000	3.615176000	2.654596000				
H	7.318173000	2.857097000	0.222552000	H	2.952164000	3.978331000	2.138002000				
O	5.917387000	1.372567000	0.813264000	N	2.894270000	2.238715000	-2.987191000				
C	4.794022000	2.184554000	0.379530000	H	2.486577000	3.033396000	-3.487122000				
C	4.570321000	1.954489000	-1.134627000	C	3.038014000	1.016601000	-3.633642000				
C	3.646725000	1.611894000	1.244232000	H	3.459358000	0.173823000	-3.055735000				
H	4.992082000	3.256054000	0.610554000	O	2.708439000	0.942373000	-4.816653000				
C	3.164822000	2.444370000	-1.555699000	C	-0.516467000	0.574303000	-2.906760000				
H	5.370176000	2.450338000	-1.735449000	H	0.266434000	1.088370000	-3.503404000				
H	3.679694000	0.497016000	1.245798000	H	-1.297619000	0.175135000	-3.581480000				
C	2.115163000	1.715485000	-0.677523000	O	0.194207000	-0.505398000	-2.290599000				
H	3.096187000	3.552981000	-1.343385000	H	-0.441234000	-1.143975000	-1.887120000				
H	2.058100000	0.617379000	-0.810597000	O	-0.385077000	2.162009000	1.790798000				
O	0.881966000	2.381058000	-0.899061000	H	0.598615000	2.327889000	1.700224000				
C	-0.300550000	1.604558000	-0.566486000	N	-3.286687000	2.825022000	1.617373000				
C	-1.024975000	2.409056000	0.528859000	H	-2.891741000	2.801095000	2.570598000				
C	-1.120669000	1.532504000	-1.866930000	C	-3.397140000	4.241491000	1.162501000				
H	-0.024511000	0.586798000	-0.203955000	H	-3.215717000	4.923800000	2.023596000				

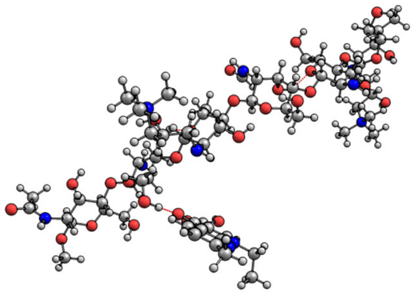

C	-2.480608000	1.926955000	0.750183000	H	-2.642504000	4.520909000	0.387678000
H	-0.964685000	3.503562000	0.315563000	C	-4.814176000	4.452847000	0.588863000
H	-1.280977000	2.544193000	-2.308306000	H	-5.479999000	3.576248000	0.848750000
C	-3.221894000	1.710077000	-0.590447000	C	-5.521058000	5.735438000	1.081751000
H	-2.434325000	0.931311000	1.286194000	H	-5.363488000	5.815684000	2.188599000
H	-3.604605000	2.621571000	-1.096092000	H	-6.627497000	5.597756000	0.969531000
O	-4.231361000	0.758584000	-0.286142000	O	-4.627010000	4.469653000	-0.835294000
C	-5.350556000	0.663407000	-1.208872000	H	-5.488571000	4.341836000	-1.305162000
C	-5.035435000	-0.465349000	-2.215234000	N	-5.139891000	7.057099000	0.423240000
C	-6.516717000	0.289127000	-0.267472000	C	-7.019247000	1.474646000	0.567916000
H	-5.514852000	1.627616000	-1.735093000	H	-6.195301000	2.197015000	0.784563000
C	-6.292703000	-1.055049000	-2.907858000	H	-7.858521000	2.002775000	0.072097000
H	-4.420746000	-1.251575000	-1.717613000	O	-7.577176000	1.034186000	1.806461000
H	-6.231862000	-0.569680000	0.392940000	H	-6.955690000	0.376638000	2.261956000
C	-7.436187000	-1.275387000	-1.892157000	O	-4.287666000	0.073032000	-3.314452000
H	-6.644117000	-0.320080000	-3.687386000	H	-3.421066000	0.458268000	-2.978346000
H	-7.335360000	-2.156719000	-1.226812000	N	-6.019421000	-2.348042000	-3.584152000
O	-8.633012000	-1.298658000	-2.666283000	H	-5.389035000	-2.198102000	-4.374081000
C	-9.775888000	-1.912813000	-2.015695000	H	-5.548554000	-3.002103000	-2.957965000
C	-10.970405000	-1.076245000	-2.536329000	C	-8.868118000	-4.318705000	-1.787041000
C	-9.844248000	-3.376696000	-2.507999000	H	-7.862400000	-4.289916000	-2.253146000
H	-9.682270000	-1.849699000	-0.908860000	H	-9.252708000	-5.357498000	-1.789907000

Table S4. Structures and Cartesian coordinates of minimum energy structures for the interaction between the poly(4-vinylbenzyl) trimethylammonium chloride (polymer-2) and the nadilixic acid (antibiotic) at PM6//B3LYP/6-31g(d,p) identified by Kick-Fukui method.

**Structure 1: I2-E1 (241 atoms)**											
C	-15.931757000	2.372462000	1.006821000	C	2.561220000	-6.340151000	0.440105000	H	10.907699000	-3.621234000	4.552618000
C	-15.418197000	1.168508000	1.655935000	C	4.060033000	-4.892093000	2.301132000	H	9.332065000	-3.428158000	5.354139000
C	-15.972627000	0.912841000	3.000898000	H	4.371421000	-3.459377000	0.721735000	H	9.587170000	-4.760785000	4.198145000
C	-16.904252000	1.785353000	3.604254000	C	2.709974000	-6.824546000	1.738786000	C	9.703473000	-1.371535000	3.594318000
C	-16.840848000	3.204579000	1.647972000	H	1.978979000	-6.912643000	-0.283928000	H	9.422956000	-0.682902000	2.775008000
H	-14.853478000	-0.920241000	3.289427000	C	3.463750000	-6.104843000	2.679164000	H	9.196917000	-1.026141000	4.514276000
C	-15.576342000	-0.229087000	3.738065000	H	4.643008000	-4.317329000	3.023311000	H	10.791547000	-1.245453000	3.756675000
H	-17.202072000	4.125088000	1.140997000	H	2.227498000	-7.762589000	2.019640000	C	12.907678000	2.407605000	3.973760000
C	-17.034863000	0.466621000	5.540048000	C	0.988886000	-0.459771000	-2.958289000	H	12.884078000	3.067925000	4.860948000

C	-16.099770000	-0.455756000	4.996184000	C	0.183286000	-0.988250000	-3.980545000	H	12.499990000	1.425213000	4.279682000
H	-15.806937000	-1.331011000	5.578324000	C	0.456129000	0.529658000	-2.115144000	H	13.972554000	2.250291000	3.716251000
N	-17.431172000	1.572203000	4.854986000	C	-1.126641000	-0.524784000	-4.163022000	C	12.210584000	2.055204000	1.637574000
N	-17.342995000	2.944412000	2.894786000	C	-0.853116000	0.978547000	-2.288429000	H	11.764558000	1.071819000	1.874580000
C	-18.291786000	3.923952000	3.529809000	H	1.072615000	0.941337000	-1.309339000	H	11.677562000	2.459130000	0.755808000
H	-18.943015000	3.379741000	4.256519000	C	-1.651090000	0.456208000	-3.311011000	H	13.257022000	1.873323000	1.327591000
H	-18.972308000	4.322342000	2.740840000	H	-1.258671000	1.742823000	-1.623009000	C	12.761429000	4.320948000	2.432395000
C	-17.527388000	5.043527000	4.216575000	C	6.120023000	-0.608286000	-0.078849000	H	12.252272000	4.778572000	1.563244000
H	-18.223994000	5.737253000	4.711594000	C	6.525305000	-1.934555000	-0.308387000	H	12.724223000	5.047728000	3.264999000
H	-16.846508000	4.669244000	4.993252000	C	6.273605000	-0.068161000	1.208651000	H	13.826208000	4.201085000	2.154728000
H	-16.927793000	5.640287000	3.516830000	C	7.086746000	-2.693538000	0.719108000	C	2.936232000	8.958362000	-1.304662000
O	-14.586712000	0.415320000	1.162253000	H	6.406609000	-2.378085000	-1.298092000	H	3.266174000	8.029741000	-0.799188000
C	-15.484662000	2.718314000	-0.332978000	C	6.823009000	-0.826532000	2.240537000	H	3.348112000	8.945950000	-2.330902000
O	-14.965662000	2.051816000	-1.208448000	H	5.945563000	0.960757000	1.403156000	H	3.410695000	9.805093000	-0.772041000
O	-15.733958000	4.052233000	-0.603581000	C	7.250891000	-2.141167000	1.998487000	C	0.925006000	9.086781000	0.113423000
H	-15.455880000	4.319960000	-1.524863000	H	7.393945000	-3.722424000	0.520219000	H	-0.177392000	9.160905000	0.155683000
C	-17.619141000	0.257382000	6.889618000	H	6.913821000	-0.391289000	3.237577000	H	1.219072000	8.166610000	0.653766000
H	-18.123185000	-0.721567000	6.965290000	C	2.458788000	4.564699000	-1.442484000	H	1.331175000	9.941525000	0.687058000
H	-16.840118000	0.270413000	7.671614000	C	2.206591000	5.849327000	-1.950996000	C	1.044985000	10.386927000	-1.976460000
H	-18.363016000	1.021001000	7.169195000	C	1.570882000	4.019590000	-0.499918000	H	1.485525000	11.257398000	-1.454120000
C	-0.957837000	-6.523811000	-1.345849000	C	1.085198000	6.572828000	-1.522873000	H	1.393025000	10.429685000	-3.025861000
H	-1.856623000	-6.022716000	-0.968265000	H	2.883259000	6.278681000	-2.691065000	H	-0.051531000	10.535279000	-1.985522000
H	-0.476030000	-7.013261000	-0.491965000	C	0.451973000	4.738147000	-0.079616000	C	-2.247712000	1.056807000	-6.449203000
H	-1.291249000	-7.313460000	-2.029112000	H	1.769537000	3.024310000	-0.086118000	H	-3.270933000	1.179638000	-6.048124000
C	-0.007571000	-5.545554000	-2.043329000	C	0.199616000	6.014630000	-0.591260000	H	-1.556894000	1.590992000	-5.769247000
H	0.915477000	-6.112802000	-2.334306000	H	-0.233079000	4.302183000	0.649693000	H	-2.213898000	1.581065000	-7.423322000
C	0.405861000	-4.410148000	-1.085870000	C	8.068125000	4.704471000	0.158918000	C	-2.824363000	-1.059334000	-7.568178000

H	-0.416790000	-3.671665000	-1.009097000
H	0.519773000	-4.806426000	-0.058524000
C	1.703803000	-3.699777000	-1.520474000
H	1.625304000	-3.483869000	-2.618923000
C	1.841147000	-2.355478000	-0.778343000
H	2.120166000	-2.542533000	0.277054000
H	0.852658000	-1.856542000	-0.729334000
C	2.863640000	-1.400124000	-1.427071000
H	3.822267000	-1.963955000	-1.577687000
C	3.159101000	-0.232862000	-0.461249000
H	3.530569000	-0.638974000	0.499870000
H	2.205738000	0.284905000	-0.214955000
C	4.160976000	0.799504000	-1.012048000
H	3.799913000	1.106635000	-2.036613000
C	4.168755000	2.060319000	-0.124400000
H	4.804418000	1.886576000	0.771684000
H	3.150580000	2.242671000	0.284382000
C	4.653924000	3.319906000	-0.871780000
H	5.595565000	3.050766000	-1.420633000
C	4.993412000	4.431226000	0.139813000
H	5.591534000	4.007019000	0.970626000
H	4.060250000	4.796973000	0.612661000
C	5.755321000	5.618090000	-0.483566000
H	5.206800000	5.942163000	-1.405808000
C	-0.622028000	-4.999627000	-3.356607000
H	0.019432000	-4.177102000	-3.741299000
H	-0.559498000	-5.792957000	-4.131206000
C	8.199723000	3.321912000	0.374215000
C	8.800296000	5.589633000	0.968230000
C	9.050567000	2.835326000	1.366720000
H	7.632900000	2.619450000	-0.237994000
C	9.643584000	5.105981000	1.968612000
H	8.707477000	6.666665000	0.816256000
C	9.786951000	3.723907000	2.165419000
H	10.186178000	5.812537000	2.599331000
H	-5.722453000	-3.269854000	-2.897915000
H	-2.674576000	0.810978000	-3.432623000
H	-0.685940000	6.561416000	-0.267211000
C	10.677760000	3.197292000	3.254972000
H	10.661402000	3.883343000	4.138801000
H	10.288513000	2.220241000	3.639057000
C	7.832518000	-2.967549000	3.110075000
H	7.342985000	-2.712575000	4.083891000
H	7.609608000	-4.051660000	2.945967000
C	3.572422000	-6.594518000	4.095784000
H	2.622323000	-7.098678000	4.405335000
H	3.684885000	-5.729866000	4.797975000
C	0.799814000	7.933284000	-2.104044000
H	1.159038000	7.987687000	-3.163099000
H	-0.305490000	8.102923000	-2.166012000
C	-1.992697000	-1.120586000	-5.242925000
H	-1.742455000	-2.202576000	-5.387615000
H	-3.066509000	-1.114873000	-4.924071000
C	-5.556461000	-5.771052000	-3.989898000
H	-2.775414000	-0.583008000	-8.566067000
H	-2.590965000	-2.131392000	-7.708349000
H	-3.875327000	-0.991255000	-7.229078000
H	0.582612000	-1.766261000	-4.632425000
C	-6.604066000	-5.951360000	-1.725742000
H	-7.410395000	-5.251540000	-2.014139000
H	-7.017809000	-6.623255000	-0.949768000
H	-5.803607000	-5.357202000	-1.244780000
C	-7.219329000	-7.534954000	-3.509266000
H	-7.653197000	-8.234913000	-2.769985000
H	-8.043372000	-6.887862000	-3.863776000
H	-6.882675000	-8.143105000	-4.369949000
C	-4.996871000	-7.670730000	-2.459054000
H	-4.572164000	-8.243689000	-3.304199000
H	-4.164202000	-7.129686000	-1.969743000
H	-5.377205000	-8.406899000	-1.725224000
C	-0.465569000	-0.506852000	-7.126196000
H	-0.148365000	-1.560383000	-7.238456000
H	-0.372120000	-0.031478000	-8.121283000
H	0.263117000	-0.006268000	-6.460177000
H	9.135742000	1.757989000	1.519061000

C	2.955588000	-4.592660000	-1.333944000	H	-5.235346000	-6.370403000	-4.879354000	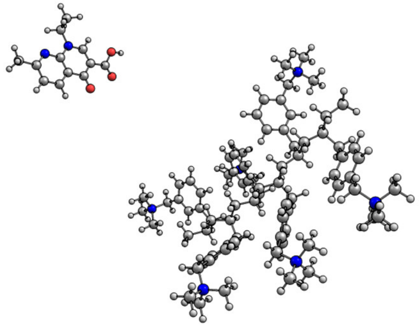
H	3.851943000	-4.015814000	-1.649702000	H	-6.404556000	-5.139828000	-4.358867000	
H	2.894545000	-5.439750000	-2.051158000	N	12.135417000	3.000030000	2.813847000	
C	2.411653000	-0.921459000	-2.826685000	N	4.739707000	-7.564130000	4.327805000	
H	3.077420000	-0.085230000	-3.152472000	N	9.351556000	-2.802863000	3.264362000	
H	2.616832000	-1.739869000	-3.550586000	N	1.430496000	9.083043000	-1.310273000	
C	5.577572000	0.215101000	-1.211190000	N	-6.085640000	-6.727143000	-2.914047000	
H	5.574940000	-0.383138000	-2.148685000	N	-1.874345000	-0.400424000	-6.590690000	
H	6.285700000	1.049495000	-1.417559000	C	6.289068000	8.093266000	-0.118660000	
C	3.638198000	3.781310000	-1.944120000	H	7.363575000	8.029868000	-0.331207000	
H	3.264913000	2.884430000	-2.492327000	H	5.790102000	8.331594000	-1.064804000	
H	4.188548000	4.367083000	-2.711734000	H	6.142164000	8.947270000	0.553204000	
C	5.757008000	6.805234000	0.502784000	C	4.586352000	-8.800651000	3.473533000	
H	6.347469000	6.548654000	1.403982000	H	3.630680000	-9.319759000	3.672916000	
H	4.724496000	6.974806000	0.870382000	H	4.620317000	-8.557763000	2.394538000	
C	7.180873000	5.222637000	-0.935617000	H	5.397391000	-9.529496000	3.663638000	
H	7.095480000	4.467019000	-1.746622000	C	6.053653000	-6.892657000	4.004361000	
H	7.656017000	6.103432000	-1.419581000	H	6.107478000	-6.599912000	2.938536000	
C	-2.039009000	-4.522374000	-3.218075000	H	6.206440000	-5.981970000	4.612614000	
C	-3.101252000	-5.339330000	-3.638119000	H	6.913444000	-7.561450000	4.200796000	
C	-2.320580000	-3.255241000	-2.681967000	C	4.735103000	-7.971110000	5.786272000	
C	-4.424914000	-4.898758000	-3.512040000	H	5.567942000	-8.661460000	6.019838000	
H	-2.887237000	-6.318606000	-4.068429000	H	4.842400000	-7.098261000	6.456880000	
C	-3.640015000	-2.814021000	-2.568413000	H	3.797977000	-8.489782000	6.063288000	
H	-1.501332000	-2.612921000	-2.349978000	C	10.059156000	-3.209146000	1.992761000	
C	-4.696911000	-3.629345000	-2.982721000	H	9.829680000	-4.253639000	1.712625000	
H	-3.848972000	-1.825754000	-2.154337000	H	9.770212000	-2.563563000	1.141537000	

C	3.142579000	-5.117392000	0.060405000	H	11.158336000	-3.131523000	2.096016000				
C	3.901913000	-4.404509000	1.002803000	C	9.814879000	-3.696211000	4.395506000				
**Structure 2: I2-E2 (241 atoms)**											
C	-6.735645000	-2.732584000	-1.791692000	C	-2.591505000	1.715983000	2.256537000	H	11.538016000	-5.541712000	1.997938000
C	-6.409652000	-3.889237000	-0.959834000	C	-1.866097000	0.272026000	0.453690000	C	0.132761000	-8.698261000	-0.686338000
C	-7.583303000	-4.625956000	-0.446326000	C	-3.923629000	1.539467000	1.858802000	H	0.717212000	-7.770476000	-0.842102000
C	-8.904852000	-4.242334000	-0.761256000	C	-3.191166000	0.111938000	0.049231000	H	-0.011866000	-8.825138000	0.402958000
C	-8.054326000	-2.399798000	-2.075608000	H	-1.060889000	-0.221678000	-0.099701000	H	0.762115000	-9.541261000	-1.030645000
H	-6.396155000	-6.071411000	0.646716000	C	-4.226116000	0.741626000	0.747516000	C	-0.925688000	-8.478303000	-2.901068000
C	-7.413957000	-5.755103000	0.390131000	H	-3.426433000	-0.512361000	-0.819018000	H	-1.866817000	-8.420779000	-3.478530000
H	-8.278062000	-1.520053000	-2.717065000	C	4.182229000	0.358445000	1.191712000	H	-0.349582000	-7.555726000	-3.106545000
C	-9.820447000	-5.991387000	0.505390000	C	4.570458000	1.552289000	1.824486000	H	-0.342257000	-9.322576000	-3.316072000
C	-8.518987000	-6.434692000	0.865727000	C	4.877514000	-0.055583000	0.043629000	C	-1.915324000	-9.963979000	-1.203456000
H	-8.407186000	-7.306018000	1.512906000	C	5.640533000	2.300767000	1.333453000	H	-1.320654000	-10.826023000	-1.561820000
N	-10.012114000	-4.910815000	-0.297949000	H	4.038813000	1.898125000	2.712198000	H	-2.128504000	-10.140702000	-0.132374000
N	-9.124691000	-3.114107000	-1.608925000	C	5.943081000	0.692552000	-0.453771000	H	-2.881570000	-9.991073000	-1.741904000
C	-10.518558000	-2.650410000	-1.930946000	H	4.568758000	-0.974246000	-0.470167000	C	-6.093499000	0.109803000	3.337167000
H	-11.209460000	-3.527357000	-1.899939000	C	6.345058000	1.868021000	0.199623000	H	-6.879905000	0.209088000	2.566371000
H	-10.535509000	-2.280276000	-2.983161000	H	5.923918000	3.226820000	1.838100000	H	-5.284579000	-0.514900000	2.912486000
C	-10.962744000	-1.570440000	-0.958557000	H	6.460400000	0.358384000	-1.355204000	H	-6.532152000	-0.458353000	4.179887000
H	-11.995844000	-1.258515000	-1.174540000	C	-0.111815000	-4.314254000	-0.205251000	C	-6.722701000	2.242852000	4.393026000
H	-10.945325000	-1.914471000	0.084503000	C	-0.657553000	-5.600006000	-0.058678000	H	-7.162730000	1.717334000	5.262181000
H	-10.340057000	-0.667991000	-1.013210000	C	-0.380490000	-3.590521000	-1.379331000	H	-6.394658000	3.237616000	4.749199000
O	-5.270763000	-4.253705000	-0.694212000	C	-1.453644000	-6.152091000	-1.071576000	H	-7.539818000	2.403562000	3.665106000
C	-5.665579000	-1.913516000	-2.333646000	H	-0.466069000	-6.166179000	0.854125000	H	-2.356104000	2.355825000	3.108112000
O	-4.466671000	-1.908858000	-2.124811000	C	-1.176561000	-4.139541000	-2.384130000	C	-6.629139000	8.167953000	-2.041443000
O	-6.158599000	-0.987086000	-3.237343000	H	0.051401000	-2.590536000	-1.504793000	H	-7.608808000	7.673306000	-2.174690000

H	-5.444251000	-0.400139000	-3.615575000	C	-1.721897000	-5.418541000	-2.234956000	H	-6.521363000	8.904578000	-2.860220000
C	-11.033108000	-6.690058000	1.003255000	H	-1.375390000	-3.569840000	-3.293430000	H	-5.845595000	7.400620000	-2.190916000
H	-10.944465000	-7.785521000	0.913877000	C	5.529285000	-5.102262000	0.968699000	C	-7.581764000	9.890553000	-0.560149000
H	-11.204189000	-6.474355000	2.073172000	C	5.911435000	-3.753399000	1.061547000	H	-7.484210000	10.659193000	-1.350543000
H	-11.954644000	-6.398135000	0.473669000	C	6.453025000	-6.037832000	0.472133000	H	-8.599171000	9.464428000	-0.646595000
C	-1.455177000	7.522591000	0.114560000	C	7.191522000	-3.351146000	0.679425000	H	-7.525102000	10.415758000	0.411966000
H	-2.189572000	7.263996000	-0.656896000	H	5.204979000	-3.011107000	1.435532000	C	-5.155297000	9.472246000	-0.557750000
H	-0.572253000	7.919839000	-0.398937000	C	7.728403000	-5.636340000	0.076696000	H	-5.020476000	9.949274000	0.430532000
H	-1.882127000	8.338911000	0.708387000	H	6.166023000	-7.090110000	0.391162000	H	-4.338137000	8.737113000	-0.689981000
C	-1.099692000	6.318315000	0.992427000	C	8.113056000	-4.291367000	0.193149000	H	-5.006798000	10.259514000	-1.321225000
H	-0.286984000	6.636024000	1.697145000	H	8.421797000	-6.376448000	-0.327419000	C	-4.507667000	1.259171000	4.833327000
C	-0.566426000	5.156385000	0.130810000	H	-6.956893000	5.377674000	-0.826760000	H	-4.105534000	2.222940000	5.197401000
H	-1.415586000	4.629363000	-0.347114000	H	-5.257139000	0.612549000	0.419012000	H	-4.897062000	0.715281000	5.715212000
H	0.023218000	5.555644000	-0.716879000	H	-2.351427000	-5.832858000	-3.022316000	H	-3.657837000	0.667957000	4.441411000
C	0.285547000	4.153084000	0.934128000	C	9.481047000	-3.848371000	-0.243283000	H	7.468702000	-2.298432000	0.758634000
H	-0.258286000	3.926223000	1.888806000	H	9.817000000	-4.435199000	-1.134907000				
C	0.432318000	2.836848000	0.144720000	H	9.454567000	-2.784116000	-0.590273000				
H	1.127498000	2.990236000	-0.703680000	C	7.487204000	2.683615000	-0.335902000				
H	-0.536960000	2.574710000	-0.324130000	H	7.520484000	2.625747000	-1.453350000				
C	0.918258000	1.658015000	1.013051000	H	7.331835000	3.767318000	-0.105449000				
H	1.812404000	2.003480000	1.597703000	C	4.705316000	6.742382000	-3.201159000				
C	1.381004000	0.504070000	0.098807000	H	4.079417000	7.487314000	-3.754837000				
H	2.185307000	0.871851000	-0.568627000	H	4.876429000	5.908346000	-3.927980000				
H	0.543473000	0.214317000	-0.573473000	C	-2.062259000	-7.518383000	-0.887723000				
C	1.867651000	-0.748336000	0.852117000	H	-2.273871000	-7.706121000	0.195775000				
H	1.027945000	-1.085971000	1.523709000	H	-3.062553000	-7.568551000	-1.388430000				
C	2.154923000	-1.887372000	-0.147584000	C	-5.029470000	2.250270000	2.595368000				

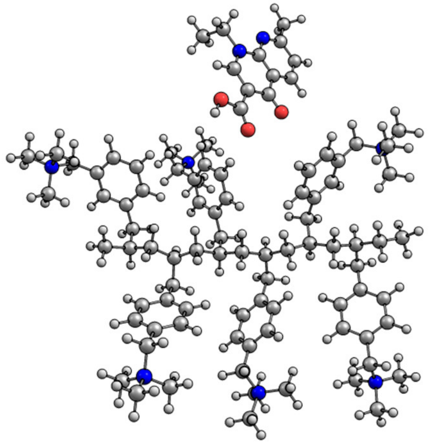

H	3.134880000	-1.711391000	-0.643328000	H	-4.673795000	3.248600000	2.957630000	
H	1.410897000	-1.864226000	-0.973935000	H	-5.874481000	2.481379000	1.897586000	
C	2.145565000	-3.281882000	0.513243000	C	-6.729033000	7.794833000	0.430176000	
H	2.728561000	-3.211374000	1.470931000	H	-6.701672000	8.321823000	1.417629000	
C	2.855968000	-4.300338000	-0.398654000	H	-7.769512000	7.389219000	0.349086000	
H	3.817031000	-3.874644000	-0.750225000	N	10.543411000	-3.977380000	0.858044000	
H	2.255789000	-4.456240000	-1.316916000	N	6.058110000	7.389562000	-2.872880000	
C	3.119752000	-5.658361000	0.283311000	N	8.854579000	2.261237000	0.220708000	
H	2.167734000	-6.003757000	0.763272000	N	-1.185498000	-8.655968000	-1.423263000	
C	-2.299938000	5.883424000	1.870511000	N	-6.514161000	8.824485000	-0.685472000	
H	-2.049991000	4.926169000	2.376925000	N	-5.578938000	1.457491000	3.786880000	
H	-2.417977000	6.617315000	2.695665000	C	3.672283000	-8.108824000	-0.220114000	
C	1.662304000	4.738492000	1.337048000	H	4.596623000	-8.202742000	0.367010000	
H	2.231825000	3.956960000	1.886406000	H	2.843273000	-8.389931000	0.439413000	
H	1.497801000	5.547772000	2.080823000	H	3.721735000	-8.855350000	-1.021575000	
C	-0.139987000	1.221304000	2.052303000	C	5.865142000	8.610050000	-2.003343000	
H	0.171079000	0.246940000	2.499187000	H	5.201202000	9.353115000	-2.482765000	
H	-0.102397000	1.940151000	2.899876000	H	5.421406000	8.347612000	-1.024168000	
C	3.075353000	-0.470711000	1.775841000	H	6.826319000	9.117459000	-1.794278000	
H	2.696871000	0.002626000	2.708135000	C	6.957777000	6.406786000	-2.161597000	
H	3.499024000	-1.444617000	2.113579000	H	6.532141000	6.099241000	-1.187748000	
C	0.713428000	-3.722044000	0.901232000	H	7.119394000	5.490663000	-2.760297000	
H	0.170397000	-2.845275000	1.324572000	H	7.955056000	6.840644000	-1.957352000	
H	0.788465000	-4.436873000	1.748949000	C	6.713366000	7.808530000	-4.171951000	
C	3.509717000	-6.700903000	-0.785962000	H	7.686976000	8.304984000	-3.996286000	
H	4.444504000	-6.390414000	-1.293374000	H	6.907299000	6.941970000	-4.831410000	
H	2.736245000	-6.710077000	-1.580700000	H	6.084852000	8.521491000	-4.737494000	

C	4.165471000	-5.541136000	1.416683000	C	8.884223000	2.415964000	1.723541000				
H	3.772403000	-4.841727000	2.186691000	H	8.664315000	3.454509000	2.032450000				
H	4.238173000	-6.520685000	1.937023000	H	8.143387000	1.757100000	2.215756000				
C	-3.590229000	5.743973000	1.115250000	H	9.875743000	2.154476000	2.139818000				
C	-4.523785000	6.793227000	1.116564000	C	9.912960000	3.155533000	-0.389296000				
C	-3.885446000	4.565857000	0.410171000	H	10.925654000	2.894007000	-0.027845000				
C	-5.725864000	6.671205000	0.408160000	H	9.931588000	3.075641000	-1.492676000				
H	-4.306333000	7.703017000	1.678011000	H	9.744124000	4.220069000	-0.139636000				
C	-5.090836000	4.441663000	-0.282979000	C	9.158384000	0.825687000	-0.137775000				
H	-3.166833000	3.743140000	0.402419000	H	8.436383000	0.128837000	0.327670000				
C	-6.015821000	5.489340000	-0.288172000	H	9.129076000	0.659805000	-1.230378000				
H	-5.313173000	3.521007000	-0.825694000	H	10.165154000	0.523612000	0.209942000				
C	2.476121000	5.258069000	0.187067000	C	11.853830000	-3.439495000	0.324091000				
C	3.368882000	4.419519000	-0.500631000	H	12.193447000	-3.997575000	-0.568681000				
C	2.355994000	6.599852000	-0.215916000	H	11.773952000	-2.373594000	0.037430000				
C	4.105025000	4.903502000	-1.583269000	H	12.660349000	-3.512369000	1.078729000				
H	3.488069000	3.379245000	-0.190068000	C	10.144500000	-3.180317000	2.077870000				
C	3.086328000	7.084059000	-1.300288000	H	10.003568000	-2.110068000	1.839522000				
H	1.677486000	7.268052000	0.317080000	H	9.198055000	-3.551316000	2.515222000				
C	3.967419000	6.238715000	-1.992624000	H	10.913842000	-3.239444000	2.871095000				
H	4.781612000	4.231990000	-2.115574000	C	10.731361000	-5.424674000	1.249156000				
H	2.960195000	8.122814000	-1.611340000	H	9.813208000	-5.848518000	1.698059000				
C	-1.551640000	1.076383000	1.561873000	H	11.002760000	-6.052016000	0.379934000				
**Structure 3: I2-E3 (241 atoms)**											
C	9.871848000	-3.501638000	0.133017000	C	2.833020000	0.538376000	-0.818755000	H	-14.475078000	-1.749473000	-0.378003000
C	9.968099000	-2.758729000	-1.117530000	C	1.932169000	-0.093896000	1.337449000	C	-3.632900000	-7.671696000	-4.010320000
C	11.193942000	-3.000076000	-1.890757000	C	4.140043000	0.411535000	-0.317559000	H	-3.976960000	-7.090327000	-3.133364000

C	12.196108000	-3.887783000	-1.438659000	C	3.229054000	-0.221659000	1.835143000	H	-3.627751000	-6.990811000	-4.881656000
C	10.894832000	-4.363164000	0.538461000	H	1.071904000	-0.294721000	1.981978000	H	-4.397221000	-8.448154000	-4.213371000
H	10.669703000	-1.655833000	-3.504384000	C	4.333545000	0.036466000	1.016583000	C	-2.352365000	-9.250931000	-2.612745000
C	11.428435000	-2.348182000	-3.128999000	H	3.389020000	-0.525836000	2.871270000	H	-1.371318000	-9.715268000	-2.394271000
H	10.800136000	-4.908420000	1.502419000	C	-2.933254000	-0.776897000	3.571388000	H	-2.702796000	-8.757009000	-1.686687000
C	13.554486000	-3.504205000	-3.311083000	C	-2.406270000	0.056911000	4.574345000	H	-3.054293000	-10.083460000	-2.823194000
C	12.589502000	-2.591224000	-3.832654000	C	-2.973757000	-2.162373000	3.806823000	C	-1.842028000	-9.039177000	-5.010703000
H	12.788580000	-2.097611000	-4.786169000	C	-1.958951000	-0.474288000	5.785021000	H	-2.555753000	-9.849098000	-5.268380000
N	13.350000000	-4.140602000	-2.127660000	H	-2.354416000	1.134262000	4.412334000	H	-1.773906000	-8.384204000	-5.899960000
N	12.017165000	-4.583509000	-0.197308000	C	-2.525752000	-2.696709000	5.016427000	H	-0.854427000	-9.524884000	-4.882577000
C	13.099097000	-5.491382000	0.332142000	H	-3.367372000	-2.831639000	3.040532000	C	7.387695000	0.056700000	-0.064885000
H	13.695593000	-5.885067000	-0.533211000	C	-2.030482000	-1.855972000	6.025679000	H	7.820818000	1.065190000	-0.042933000
H	12.618115000	-6.372898000	0.813361000	H	-1.549272000	0.196252000	6.541943000	H	6.863383000	-0.148789000	0.888881000
C	13.999820000	-4.745460000	1.299076000	H	-2.560976000	-3.776718000	5.167287000	H	8.238026000	-0.687223000	-0.088695000
H	14.802116000	-5.408927000	1.667024000	C	-2.347464000	-4.202457000	-1.361536000	C	7.290440000	0.131556000	-2.514550000
H	14.511257000	-3.895996000	0.820466000	C	-2.161876000	-5.070983000	-2.458759000	H	8.207811000	-0.518479000	-2.552094000
H	13.475610000	-4.366040000	2.183242000	C	-1.923506000	-4.623604000	-0.093724000	H	6.722527000	-0.082021000	-3.433055000
O	9.096062000	-1.979443000	-1.510646000	C	-1.543583000	-6.316260000	-2.291287000	H	7.645344000	1.173608000	-2.544535000
C	8.702521000	-3.347081000	0.971912000	H	-2.481631000	-4.751078000	-3.450188000	H	2.675831000	0.829401000	-1.856608000
O	7.719212000	-2.629434000	0.864102000	C	-1.321767000	-5.873785000	0.075458000	C	-1.311711000	10.245197000	-6.642709000
O	8.764379000	-4.177521000	2.075089000	H	-2.047784000	-3.968074000	0.771736000	H	-1.160989000	9.844996000	-7.663870000
H	7.981418000	-4.082195000	2.676884000	C	-1.120016000	-6.720684000	-1.016655000	H	-1.084430000	11.329526000	-6.692130000
C	14.814823000	-3.796094000	-4.032448000	H	-0.991713000	-6.188249000	1.068331000	H	-0.549498000	9.783792000	-5.986391000
H	14.630512000	-4.211410000	-5.038810000	C	-8.467121000	-3.077002000	-0.178847000	C	-3.695209000	10.699880000	-7.051234000
H	15.430812000	-2.889860000	-4.172005000	C	-9.134122000	-4.287266000	0.079852000	H	-3.515431000	11.794594000	-7.079452000
H	15.456760000	-4.528582000	-3.505975000	C	-9.081513000	-2.134935000	-1.021172000	H	-3.622723000	10.345390000	-8.098460000
C	-0.754441000	7.483279000	-0.732971000	C	-10.379197000	-4.552366000	-0.492590000	H	-4.744191000	10.560500000	-6.728161000

H	-0.019515000	7.516200000	-1.545930000	H	-8.676819000	-5.034730000	0.730194000	C	-2.852942000	10.542232000	-4.739562000
H	-0.222291000	7.716209000	0.197002000	C	-10.329660000	-2.396144000	-1.590687000	H	-3.880625000	10.418261000	-4.350813000
H	-1.469216000	8.294834000	-0.910951000	H	-8.580349000	-1.191072000	-1.238155000	H	-2.165172000	10.036042000	-4.035557000
C	-1.461739000	6.127082000	-0.647590000	C	-10.992463000	-3.606147000	-1.329033000	H	-2.624087000	11.626176000	-4.703465000
H	-2.140452000	6.159638000	0.243949000	H	-10.778346000	-1.653602000	-2.252685000	C	6.063411000	-1.615321000	-1.293448000
C	-0.440261000	4.984752000	-0.434094000	H	-0.583957000	7.423418000	-6.745117000	H	5.448107000	-1.847774000	-2.171289000
H	-0.214159000	4.506991000	-1.409668000	H	5.343398000	-0.064320000	1.429796000	H	6.978923000	-2.274468000	-1.306142000
H	0.526883000	5.409394000	-0.097884000	H	-0.612301000	-7.673813000	-0.869043000	H	5.524419000	-1.909084000	-0.375293000
C	-0.897234000	3.907608000	0.573802000	C	-12.295895000	-3.926272000	-2.010630000	H	-10.863459000	-5.509999000	-0.292270000
H	-1.915785000	3.563897000	0.256273000	H	-12.345052000	-3.416424000	-3.005711000				
C	0.053544000	2.683122000	0.538511000	H	-12.348430000	-5.020635000	-2.240959000				
H	0.583635000	2.568668000	1.502118000	C	-1.510089000	-2.434132000	7.314264000				
H	0.868692000	2.867660000	-0.190562000	H	-1.075967000	-3.449460000	7.133161000				
C	-0.649378000	1.353728000	0.169843000	H	-0.659739000	-1.816377000	7.697679000				
H	-1.459943000	1.591665000	-0.567896000	C	2.366828000	8.898571000	3.622274000				
C	-1.310598000	0.678360000	1.395934000	H	1.966576000	9.833934000	3.155021000				
H	-1.547750000	1.461825000	2.143131000	H	3.383158000	8.760643000	3.175173000				
H	-0.582495000	0.011242000	1.903253000	C	-1.237680000	-7.181217000	-3.490639000				
C	-2.609448000	-0.097583000	1.072723000	H	-1.139378000	-6.546491000	-4.406743000				
H	-3.180354000	0.509923000	0.319459000	H	-0.233232000	-7.660379000	-3.360635000				
C	-2.349147000	-1.485430000	0.448043000	C	5.287730000	0.778054000	-1.231051000				
H	-2.128535000	-2.230542000	1.236351000	H	4.906044000	0.878421000	-2.278359000				
H	-1.408486000	-1.427651000	-0.150994000	H	5.648036000	1.799066000	-0.945764000				
C	-3.493850000	-1.991003000	-0.468100000	C	-3.036588000	8.488890000	-6.153479000				
H	-3.950887000	-1.086692000	-0.955584000	H	-4.110914000	8.354449000	-5.871958000				
C	-4.616020000	-2.691683000	0.327133000	H	-2.964670000	8.118544000	-7.208600000				
H	-4.849613000	-2.052202000	1.210986000	N	-13.548554000	-3.540235000	-1.206683000				

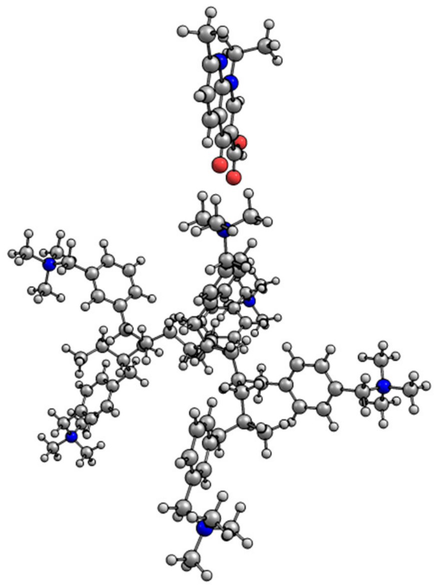

H	-4.250464000	-3.646633000	0.747054000	N	2.532767000	9.145673000	5.131071000	
C	-5.917402000	-2.944782000	-0.478725000	N	-2.563684000	-2.550916000	8.429468000	
H	-6.004337000	-2.167567000	-1.276158000	N	-2.270667000	-8.281951000	-3.770007000	
C	-2.377524000	5.896807000	-1.877300000	N	-2.714266000	9.989958000	-6.140085000	
H	-2.611388000	4.813929000	-1.965497000	N	6.492296000	-0.168512000	-1.260180000	
H	-3.356250000	6.379188000	-1.666198000	C	-6.543310000	-4.343201000	-2.523760000	
C	-1.061341000	4.457570000	2.017281000	H	-7.583277000	-3.989418000	-2.489357000	
H	-0.932407000	3.624039000	2.741016000	H	-6.009348000	-3.698098000	-3.229506000	
H	-2.121702000	4.769399000	2.141443000	H	-6.571208000	-5.351833000	-2.950721000	
C	0.330586000	0.399978000	-0.561324000	C	1.199824000	9.453963000	5.773928000	
H	-0.122557000	-0.624165000	-0.583793000	H	0.716826000	10.338062000	5.315995000	
H	0.379511000	0.710097000	-1.626635000	H	0.497099000	8.603921000	5.688118000	
C	-3.524208000	-0.187688000	2.321609000	H	1.308079000	9.678712000	6.854777000	
H	-3.914859000	0.829555000	2.541624000	C	3.142617000	7.934373000	5.799139000	
H	-4.431507000	-0.780195000	2.034610000	H	2.502806000	7.039962000	5.678798000	
C	-2.949554000	-2.850642000	-1.633665000	H	4.138572000	7.694914000	5.382443000	
H	-2.186706000	-2.249562000	-2.180960000	H	3.282170000	8.094924000	6.887402000	
H	-3.785003000	-2.974690000	-2.362980000	C	3.455139000	10.332228000	5.325097000	
C	-5.901829000	-4.341651000	-1.139152000	H	3.618068000	10.558572000	6.399519000	
H	-6.422598000	-5.073160000	-0.489267000	H	4.456223000	10.161753000	4.885055000	
H	-4.863958000	-4.719041000	-1.216113000	H	3.047950000	11.256696000	4.870940000	
C	-7.138322000	-2.788770000	0.467266000	C	-3.121254000	-1.190262000	8.780205000	
H	-7.001097000	-3.446388000	1.351462000	H	-2.329770000	-0.496396000	9.118699000	
H	-7.152550000	-1.755270000	0.874303000	H	-3.634123000	-0.724052000	7.917538000	
C	-1.847916000	6.378849000	-3.201006000	H	-3.862896000	-1.251492000	9.603285000	
C	-2.606320000	7.263210000	-3.987547000	C	-1.900125000	-3.141133000	9.657254000	
C	-0.618764000	5.909083000	-3.695007000	H	-2.613958000	-3.251082000	10.500685000	

C	-2.157753000	7.645397000	-5.261567000	H	-1.486405000	-4.149990000	9.465833000	
H	-3.560869000	7.629840000	-3.612397000	H	-1.071747000	-2.508963000	10.030524000	
C	-0.165489000	6.304892000	-4.953199000	C	-3.694190000	-3.458239000	8.000347000	
H	-0.022018000	5.224755000	-3.087460000	H	-4.223721000	-3.058983000	7.114816000	
C	-0.935326000	7.162952000	-5.746529000	H	-3.334020000	-4.473636000	7.751071000	
H	0.789304000	5.933468000	-5.332871000	H	-4.449869000	-3.577918000	8.803932000	
C	-0.158657000	5.590258000	2.424933000	C	-14.772982000	-3.910767000	-2.018909000	
C	1.226166000	5.413217000	2.580942000	H	-14.805837000	-3.383187000	-2.991464000	
C	-0.709918000	6.854373000	2.705668000	H	-14.821132000	-4.996696000	-2.230764000	
C	2.037966000	6.470808000	2.998599000	H	-15.713888000	-3.653456000	-1.489462000	
H	1.675013000	4.443089000	2.364852000	C	-13.590189000	-4.285365000	0.107940000	
C	0.099669000	7.914012000	3.114969000	H	-13.585336000	-5.381792000	-0.040372000	
H	-1.783948000	7.018228000	2.594533000	H	-12.727724000	-4.028222000	0.751419000	
C	1.483251000	7.731814000	3.270294000	H	-14.507601000	-4.048092000	0.684483000	
H	3.112264000	6.309677000	3.097472000	C	-13.568015000	-2.052417000	-0.939504000	
H	-0.354617000	8.889411000	3.298853000	H	-12.693330000	-1.734921000	-0.340777000	
C	1.721237000	0.293548000	0.004303000	H	-13.564394000	-1.466959000	-1.877771000	

polymers MDPI

Table S5. Isosurface NCI plot (0.5 a.u.) of the noncovalent interaction for polymer and antibiotics.

Polymer 1-antibiotic	
**Model**	**NCI Isosurface**
I1-E1	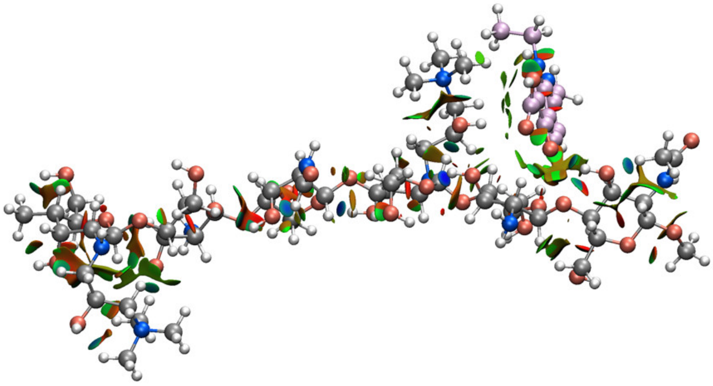
I1-E2*	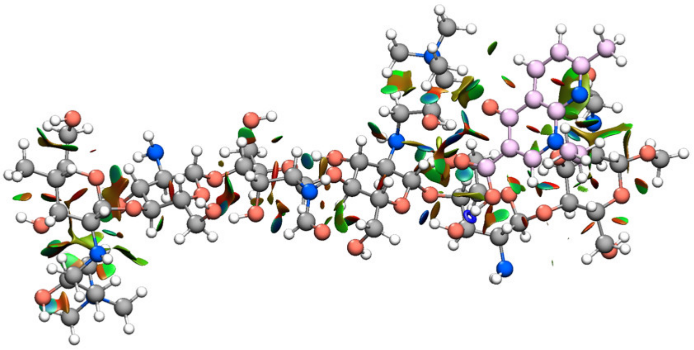
I1-E3	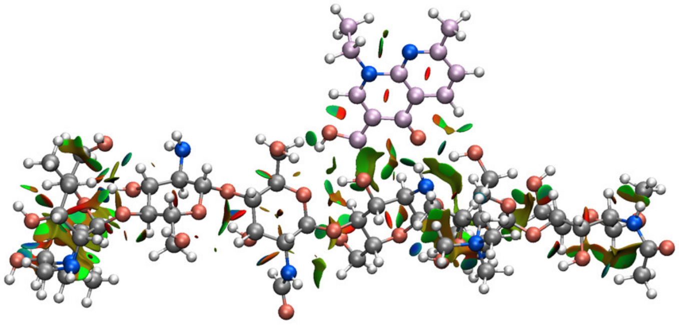
I1-E4	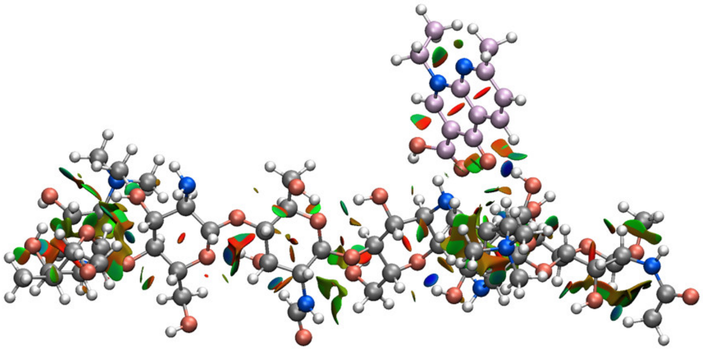
Polymer 2-antibiotic	

**Model**	
I2-E1	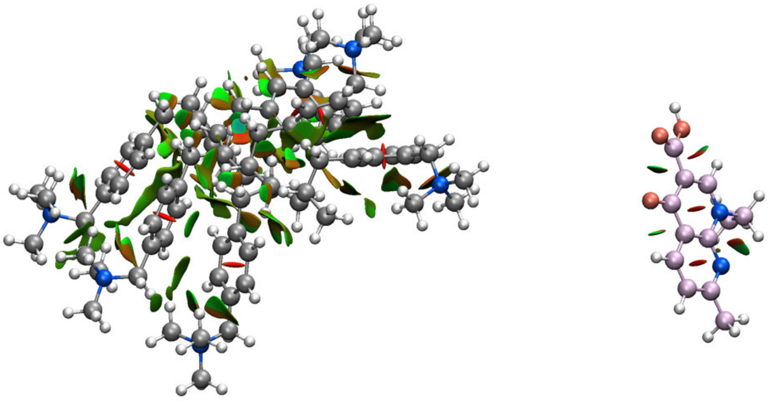
I2-E2*	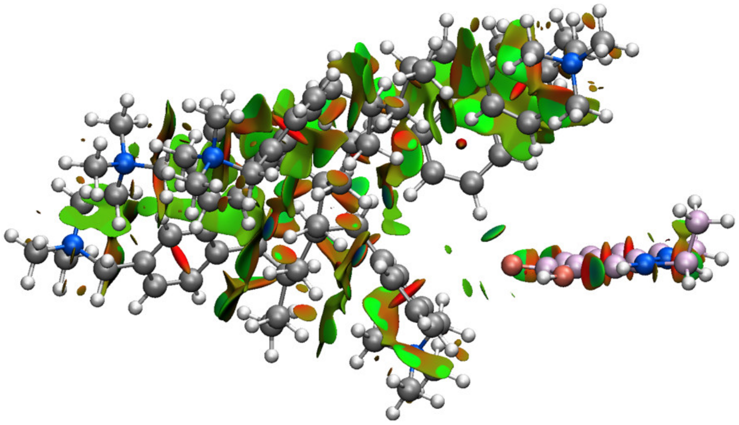
I2-E3	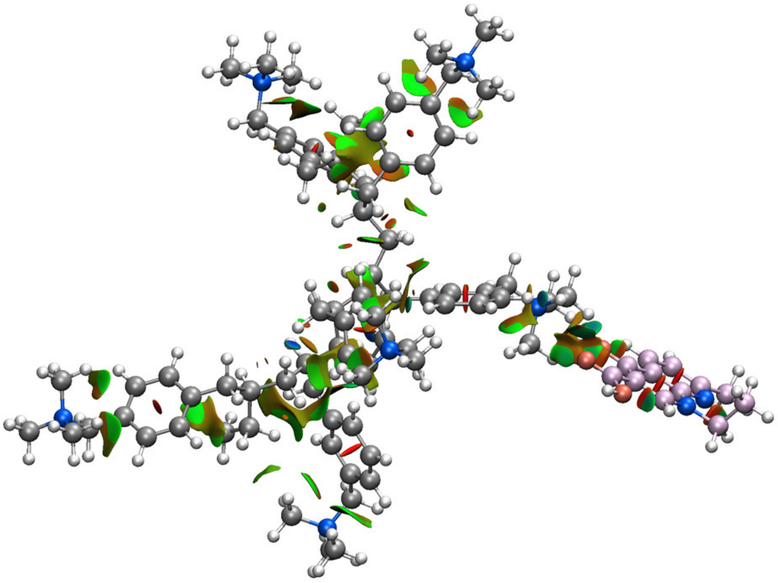

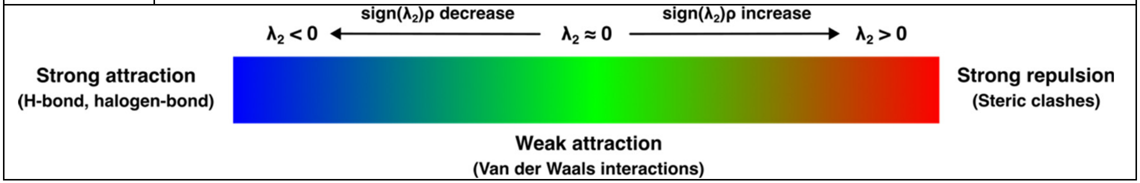

Supplement: Supplementary file 1 [file polymers-15-03185-s001.zip › polymers-2514000-supplementary.pdf]
